# A replication-deficient gammaherpesvirus vaccine protects mice from lytic disease and reduces latency establishment

**DOI:** 10.1038/s41541-024-00908-x

**Published:** 2024-06-24

**Authors:** Wesley A. Bland, Dipanwita Mitra, Shana Owens, Kyle McEvoy, Chad H. Hogan, Luciarita Boccuzzi, Varvara Kirillov, Thomas J. Meyer, Camille Khairallah, Brian S. Sheridan, J. Craig Forrest, Laurie T. Krug

**Affiliations:** 1 Department of Microbiology and Immunology, University of Arkansas for Medical Sciences, Little Rock, AR USA; 2 HIV and AIDS Malignancy Branch, National Cancer Institute, Bethesda, MD USA; 3 Department of Microbiology and Immunology, Stony Brook University, Stony Brook, NY USA; 4 Graduate Program in Genetics, Stony Brook University, Stony Brook, NY USA; 5 CCR Collaborative Bioinformatics Resource, National Cancer Institute, National Institutes of Health, Bethesda, MD USA; 6 Advanced Biomedical Computational Science, Frederick National Laboratory for Cancer Research, Frederick, MD USA; 7 Winthrop P. Rockefeller Cancer Institute, University of Arkansas for Medical Sciences, Little Rock, AR USA; 8 Present Address: Environment, Health and Safety, University of North Carolina, Chapel Hill, NC USA; 9 Present Address: Institute for Genomic Health, Icahn School of Medicine at Mount Sinai, New York, NY USA; 10 Present Address: Doctor of Medicine Program, Rush University Medical Center, 1650 West Harrison Street, Chicago, IL USA

**Keywords:** Infection, Viral infection, Live attenuated vaccines, Tumour virus infections, Live attenuated vaccines

## Abstract

Gammaherpesviruses are oncogenic viruses that establish lifelong infections and are significant causes of morbidity and mortality. Vaccine strategies to limit gammaherpesvirus infection and disease are in development, but there are no FDA-approved vaccines for Epstein-Barr or Kaposi sarcoma herpesvirus. As a new approach to gammaherpesvirus vaccination, we developed and tested a replication-deficient virus (RDV) platform, using murine gammaherpesvirus 68 (MHV68), a well-established mouse model for gammaherpesvirus pathogenesis studies and preclinical therapeutic evaluations. We employed codon-shuffling-based complementation to generate revertant-free RDV lacking expression of the essential replication and transactivator protein encoded by *ORF50* to arrest viral gene expression early after de novo infection. Inoculation with RDV-50.stop exposes the host to intact virion particles and leads to limited lytic gene expression in infected cells yet does not produce additional infectious particles. Prime-boost vaccination of mice with RDV-50.stop elicited virus-specific neutralizing antibody and effector T cell responses in the lung and spleen. In contrast to vaccination with heat-inactivated WT MHV68, vaccination with RDV-50.stop resulted in a near complete abolishment of virus replication in the lung 7 days post-challenge and reduction of latency establishment in the spleen 16 days post-challenge with WT MHV68. *Ifnar1*^*−/−*^ mice, which lack the type I interferon receptor, exhibit severe disease and high mortality upon infection with WT MHV68. RDV-50.stop vaccination of *Ifnar1*^*−/−*^ mice prevented wasting and mortality upon challenge with WT MHV68. These results demonstrate that prime-boost vaccination with a gammaherpesvirus that is unable to undergo lytic replication offers protection against acute replication, impairs the establishment of latency, and prevents severe disease upon the WT virus challenge. Our study also reveals that the ability of a gammaherpesvirus to persist in vivo despite potent pre-existing immunity is an obstacle to obtaining sterilizing immunity.

## Introduction

Preventive vaccination against hepatitis B virus^1^ and high-risk human papillomaviruses^2^ has reduced the virus-associated cancer burden; however, an effective vaccine against the oncogenic gammaherpesviruses (GHVs) has not yet been deployed. GHVs include the human pathogens Epstein-Barr virus (EBV), formally designated human herpesvirus 4 (HHV-4), and Kaposi sarcoma herpesvirus (KSHV) formally designated human herpesvirus 8 (HHV-8). EBV causes infectious mononucleosis (IM) in adolescents and young adults during primary infection and is associated with the development of multiple sclerosis (MS)^3,4^. In addition, EBV is a cause of numerous lymphomas and epithelial-derived carcinomas of the oropharynx and gastrointestinal (GI) system^5,6^. KSHV is etiologically associated with lymphoproliferative diseases including primary effusion lymphoma (PEL) and a B cell variant of multicentric Castleman disease^7^. KSHV is also the cause of Kaposi sarcoma, a tumor of presumed endothelial cell origin that manifests in the skin, lungs, or GI tract^7,8^ which is often accompanied by a presentation of inflammatory cytokine syndrome (KICS)^9^. Vaccines that block infection or lower viral burden are critical to develop, as a means to reduce the incidence of GHV-associated diseases and cancers.

While knowledge of immunodominant epitopes and correlates of immune protection for KSHV lag behind what is known for EBV, the need for a KSHV vaccine is paramount for at-risk populations^10^. People living with HIV (PLWH) and transplant patients exhibit higher EBV and/or KSHV viral loads in circulating peripheral blood mononuclear cells (PBMCs) and are at increased risk for GHV-associated diseases and cancer, even when HIV is well controlled by anti-retroviral therapy^11–13^. KSHV is typically horizontally acquired during childhood in sub-Saharan Africa^14^, and is a leading cause of morbidity and mortality in geographical areas where malaria and HIV co-infections are endemic^15,16^. Infection by KSHV elicits weaker virus-neutralizing antibody and cell-mediated immune responses compared to responses against EBV, cytomegalovirus (CMV) and influenza^17^. KSHV-specific T cell responses in immunocompetent individuals from rural Uganda are generally low and heterogenous^17^. Studies of KSHV-infected patient cohorts reveal a weak and highly disparate immune response to multiple epitopes lacking immunodominance; there is no clear latent or lytic antigen to target to prevent infection or clear infected cells^18^.

GHVs, like all members of the herpesvirus family, display a biphasic infection cycle. During productive lytic infection, a temporally regulated cascade of viral non-coding RNAs and gene products leads to the production of infectious particles capable of spreading to diverse cell types in the host. In contrast, the more immunologically silent latency phase is characterized by limited gene expression that promotes the viability of the infected host cell and maintenance of the non-integrated viral genome, termed an episome^19^. Reactivation from latency to reinitiate the lytic cycle occurs intermittently in response to unknown stimuli and facilitates persistence in cellular reservoirs within the host and transmission to new hosts^20^. A vaccine against KSHV or EBV that stimulates broad humoral and cellular immune responses against dozens of viral proteins in addition to the virion surface glycoproteins is hypothesized to have preventive and therapeutic potential. While it is generally considered desirable to block primary infection, prevention of virus-associated diseases and cancer is the most critical goal. Vaccine safety and cost-effectiveness are also important considerations^10^. Tractable animal models that recapitulate hallmarks of human GHV pathogenesis are needed to test different vaccine formulations, dosage, and routes of administration, to evaluate efficacy and define correlates of immune protection^10^.

Effective vaccine strategies have been developed against the human alphaherpesvirus varicella-zoster virus (VZV)^21,22^. Varivax, a live-attenuated VZV strain, protects against chickenpox following wild-type virus exposure. Notably, both wild-type and vaccine strains establish latent infection in dorsal root ganglia^23,24^. VZV reactivation results in shingles in individuals infected with wild-type VZV – a painful disease with potential long-term complications including ophthalmic zoster and post-herpetic neuralgia^21^. The glycoprotein subunit-based vaccine Shingrix is a therapeutic vaccine widely administered to individuals over 50 years of age. Despite being administered after a person is colonized by VZV, Shingrix is remarkably effective at preventing shingles^25–30^. Thus, while it is ideal for potential vaccine candidates to prevent infection by generating sterilizing immunity, therapeutic vaccine strategies might also protect against disease in individuals that harbor oncogenic GHVs. With regard to live-attenuated vaccines, safety concerns related to secondary mutations and reversion to virulence in immunocompromised individuals must be carefully addressed.

To date, there are no FDA-approved vaccines that protect against human GHVs. Efforts focused on developing prophylactic vaccines to prevent virus infection have been challenging due to the use of multiple glycoprotein complexes by GHVs to target different cell type-specific receptors. EBV glycoprotein gp350 binds to complement receptor CD21 on B cells^31^. In a phase 2 clinical trial the administration of a gp350 subunit vaccine reduced infectious mononucleosis by 78% in participants but had no impact on the rate at which the participants became infected with EBV^32,33^. Vaccines against EBV currently in trials present the gp350 subunit alone or in combination with glycoprotein subunits gH/gL/gp42 as a multivalent approach to broaden immunogenicity and block virus entry into B cells and epithelial cells^34^.

Due to the species-restricted nature of human GHVs, murine gammaherpesvirus 68 (MHV68), a GHV that infects murine rodents, is commonly used to study GHV pathogenesis and disease processes^35,36^. The MHV68 genome is colinear with KSHV, and the majority of open reading frames (ORFs) encode structural and functional homologs of EBV and KSHV genes^37^. Importantly, like human GHVs, MHV68 infection establishes life-long infection in B cells and promotes diseases such as lymphoproliferative disorders in immunocompromised animals^36,38^. Over a span of three decades, viral pathogenic determinants of latency and replication, cellular reservoirs of infection, and key immune responses that control MHV68 infection in mice have been carefully delineated^36^. Live-attenuated viruses that replicate in the absence of latency protect against WT virus challenge^39–41^, and transfer of serum and T cells from vaccinated mice prior to challenge reduces the frequency of latent cells that undergo explant reactivation in recipient mice^39^. Thus, MHV68 infection of mice is a tractable small-animal pathogenesis system to evaluate the effectiveness of rationally designed vaccines and to identify immune correlates of protection against pathogenic GHVs^42^.

Lytic replication and reactivation are controlled by the replication and transactivator (RTA) protein, a viral transcription factor encoded by *ORF50*^43,44^. In KSHV and MHV68, RTA promotes transcription of nearly all viral lytic genes^45^. Thus, RTA is essential for productive infection and lytic reactivation from latency. We recently developed a platform to produce high-titer, revertant-free stocks of a replication-deficient virus (RDV) with a premature translation stop codon in *ORF50*^46^. Here, we test the efficacy of this forward-engineered infectious, yet replication-deficient RDV-50.stop virus as a potential vaccine to stimulate an immune response that protects against wild-type MHV68 infection and virus-driven disease in mice. Immune correlates defined with this vaccine platform will inform KSHV and EBV vaccine design.

## Results

### Virus-specific adaptive immunity is induced by vaccination with RDV-50.stop

**Fig. 1:**
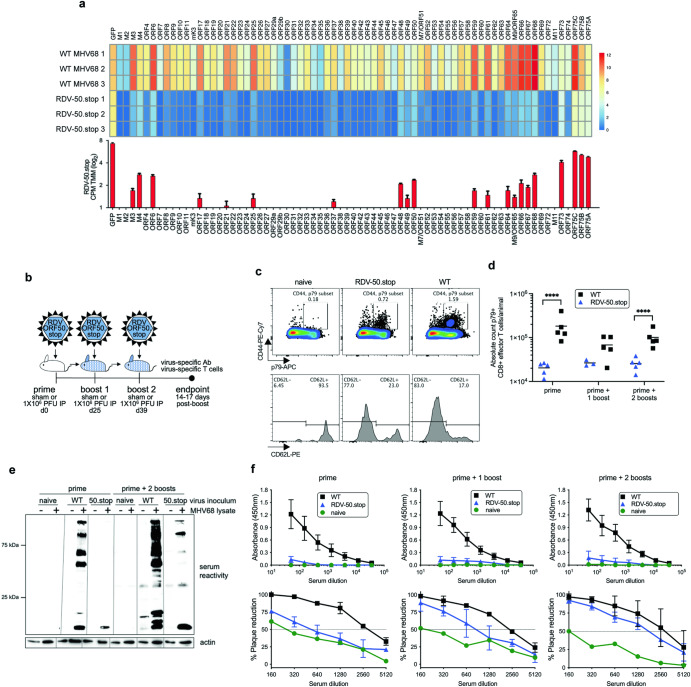
A replication-deficient recombinant MHV68 generates virus-specific immune responses upon a prime-boost regimen in C57BL/6 mice. **a** Top panel, RNAseq profile of viral gene expression in murine fibroblasts 18 hpi with RDV-50.stop or WT MHV68 (MOI 3) in biological triplicate. Scale represents log_2_ CPM TMM value for non-overlapping ORF regions. Lower panel, bars (mean +/− SD) represent ORF gene expression upon RDV-50.stop infections. **b** Schematic of prime-boost strategy to examine the immune response of C57BL/6 mice to RDV-50.stop or WT control virus infections. Naïve mice were age-matched, non-vaccinated controls. **c** Virus-specific effector CD8 T cell response based on p79 tetramer+ of CD44^hi^CD62L^−^ CD8 T cells. Representative gating strategy from naïve mice or mice infected with RDV-50.stop of WT virus at d39. **d** Total p79-reactive CD8 T cells per spleen of individual mice after initial prime and sequential boosts. Symbols represent individual mice (*N* = 3–5) and bars are mean values; ****, *p* < 0.0001 in multiple unpaired t test. **e** Immunoblot analysis of infected cell lysates testing reactivity of sera from immunized mice after initial prime or prime followed by two boosts. Lines indicate blot strips incubated with sera from indicated animals and then reassembled for imaging. **f** Top, panel defining virus-specific IgG in the sera of mice after prime and prime-boost(s). Symbols are mean values +/− SD (*N* = 5 mice). Below, neutralizing antibodies were evaluated by determining the serum dilution that led to a 50% reduction in plaque formation. Symbols are mean values +/− SD (*N* = 3 mice).

Insertion of a frameshift and translation stop codon in *ORF50* (ORF50.stop) renders MHV68 incapable of producing RTA, thereby generating RDV-50.stop^44,46^. RDV-50.stop particles undergo an abortive infection if not grown on a complementing cell line^46^. We expected that virion-associated proteins and limited RTA-independent viral gene expression would undergo antigen-presentation and promote virus-specific immune responses in vaccinated mice. Consistent with expectations, RNA-seq analysis of infected fibroblasts demonstrated that RDV-50.stop exhibits a global reduction in viral gene expression when compared to WT MHV68 (Fig. 1a). Although median expression levels were reduced ~300-fold compared to WT infection, transcripts for ORFs associated with latency (*M3*, *M4* and *ORF73*/LANA) and lytic replication that are also immunodominant epitopes (*ORF6* and *ORF61*) were detected upon infection with RDV-50.stop. Given that RDV-50.stop infection is self-limiting and viral gene expression is sharply curtailed, we hypothesized that vaccination of mice using RDV-50.stop would require multiple doses to generate a potent MHV68-specific immune response.

We tested this hypothesis using a prime-boost strategy of vaccination. C57BL/6 mice were inoculated intraperitoneally (i.p.) with 10^6^ PFU of RDV-50.stop (day 0). One experimental group received one boost at day 25 (prime + 1 boost). A separate set of animals received two boosts, one at day 25 and another on day 39 after the initial inoculation (prime + 2 boosts) (Fig. 1b). We evaluated virus-specific CD8 T cell and humoral immune responses 14 days after the final RDV-50.stop dose in comparison to mice infected with WT MHV68. CD8 T cells that are specific for lytic antigens restrict virus replication through cytolytic activity and the secretion of antiviral cytokines such as interferon-γ^47–49^. Virus-specific effector CD8 T cells (CD8 T^eff^) were identified by flow cytometry as CD19^−^, CD3^+^, CD8^+^, CD44^hi^, CD62L^−^ and tetramer-positive for the p79 immunodominant epitope derived from the ribonucleotide reductase large subunit, ORF61 (Fig. 1c) and the p56 immunodominant epitope derived from the single-stranded DNA binding protein, ORF6 (Supplementary Fig. 1). As expected, although generally lower than numbers induced by WT virus infection, virus-specific CD8 T^eff^ were elevated after vaccination with RDV-50.stop, with comparable detection following either single or double boosting regimens for p79-specific CD8 T cells (Fig. 1c, d). These findings demonstrate that sufficient viral antigen from two nonstructural proteins is generated following vaccination with a replication-deficient GHV to elicit virus-specific CD8 T^eff^ responses in mice.

Infection with MHV68 stimulates a potent polyclonal antibody response that is maintained over-time^50–52^. Passive serum transfer suggests that the antibodies generated during MHV68 infection are neutralizing and contribute to the control of WT virus infection^39,53^. To evaluate antibody responses to RDV-50.stop, we first compared lytic viral antigen reactivity in immunoblot analyses following priming alone, double boosting, and WT MHV68 infection. While a single dose of RDV-50.stop elicited minimal reactivity, the three-dose vaccination strategy stimulated the generation of antibodies that recognized many viral antigens that were also recognized by sera from animals infected with WT MHV68 (Fig. 1e). The total MHV68-specific IgG was only slightly elevated over sham-vaccination controls by ELISA, even following three doses of RDV-50.stop, and were substantially lower than IgG levels produced by WT MHV68 infection of mice (Fig. 1f, upper panel). However, 50% plaque-reduction neutralizing titer (PRNT50) assays revealed that the relatively low quantity of virus-specific IgG produced by vaccination with RDV-50.stop was capable of blocking MHV68 infection (Fig. 1f, lower panel). These findings indicate that vaccination with RDV-50.stop stimulates potent and effective neutralizing antibody production, despite eliciting MHV68-directed IgG to much lower levels than WT virus infection. Together, these data demonstrate that a prime-boost vaccination strategy using a replication-deficient GHV elicits virus-specific CD8 T^eff^ and neutralizing antibody responses in vivo.

### Vaccination with RDV-50.stop protects mice from WT challenge

**Fig. 2:**
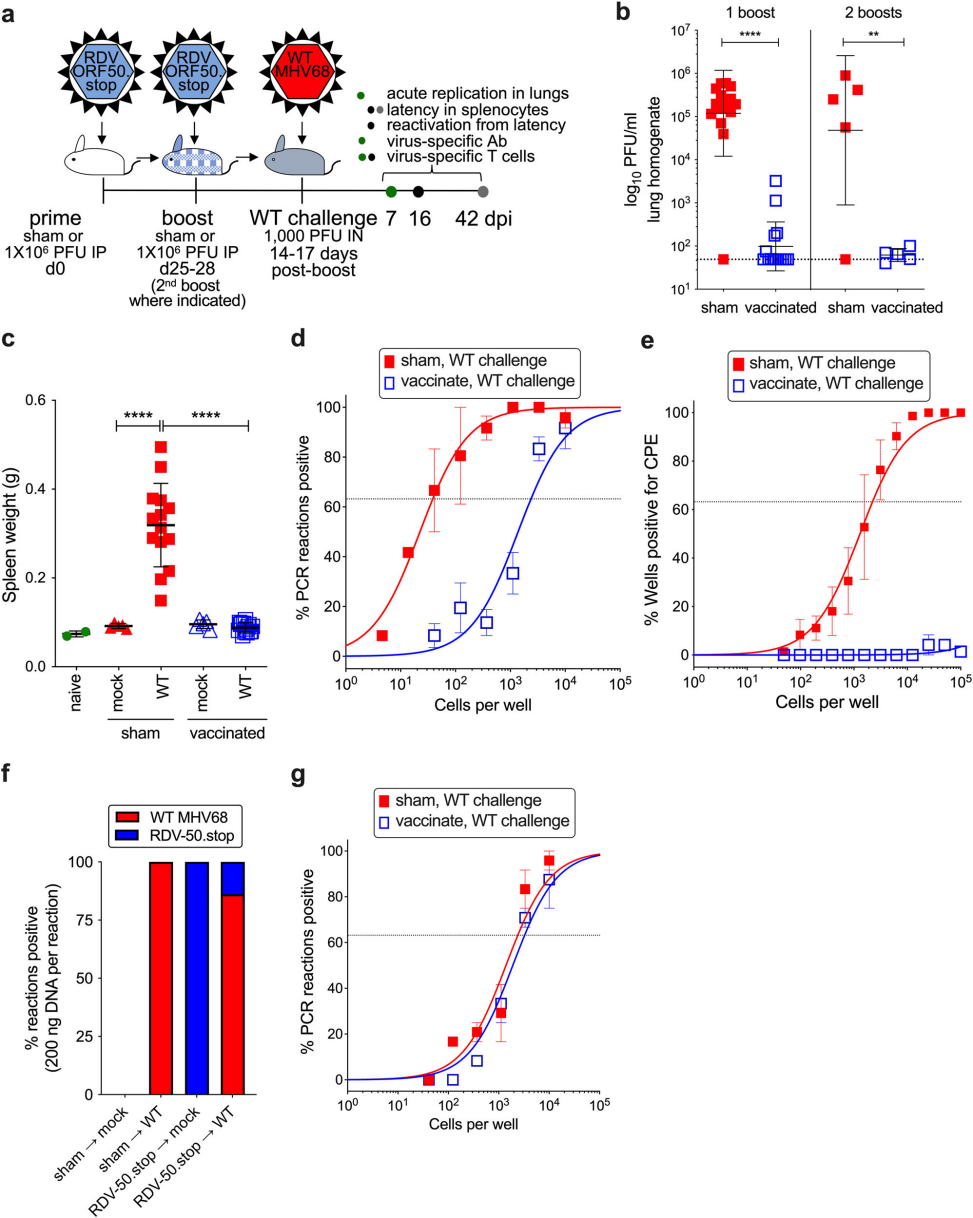
Vaccination with RDV-50.stop reduces acute replication, splenomegaly, latency and reactivation of wild-type MHV68 upon challenge in C57BL/6 mice, but does not induce sterilizing immunity. **a** C57BL/6 mice were either sham-vaccinated or vaccinated and then boosted once **b**–**e** or twice **b**, **f** with 1 × 10^6^ PFU RDV-50.stop followed by IN challenge with 1 × 10^3^ PFU WT MHV68 at d14-17 post-boost. **b** Acute replication at d7 post-challenge determined by measuring infectious particles per ml lung homogenate. Symbols denote individual mice from one (2 boosts) or three (1 boost) independent experiments for infected animals (*N* = 5 mice per experiment); bars and whiskers represent mean +/− SD. **, *p* < 0.01; ****, *p* < 0.0001 in one-tailed unpaired t-test of log-transformed values. Dotted line indicates limit of detection at 50 PFU/ml. **c** Splenomegaly determined by spleen weights. Symbols denote individual mice (*N* = 2–15); bars and whiskers represent mean +/− SD. ****, *p* < 0.0001 in Sidak’s multiple comparisons test of one-way ANOVA between the sham and vaccinated groups at d16 post-challenge. **d** The frequency of latency was determined by limiting dilution nested PCR of intact splenocytes for the viral genome at d16 post-challenge. **e** The frequency of explant reactivation determined by limiting dilution coculture of intact viable splenocytes on a monolayer of primary MEFs d16 post-challenge. Disrupted splenocytes plated in parallel did not detect preformed infectious virus in the vaccinated animals. For **d**, **e**, symbols denote the average of three experiments with five mice per experiment; error bars represent SEM. **f** PCR genotyping of a pool of splenocytes from the indicated sets of mice (*N* = 4–5) mock at d16 post-challenge. To differentiate the RDV-50.stop vaccine virus from WT challenge virus, nested PCR was performed with primers that target the FRT sequence only present within RDV-50.stop, in parallel with ‘pan-MHV68’ primers that detect both RDV-50.stop and WT MHV68. For each set, bars indicate the percentage of PCR reactions that produced RDV-50.stop FRT amplimers as a percentage of reactions that produced pan-MHV68 amplimers. The absence of RDV-50.stop FRT amplimers in samples that yielded pan-MHV68 amplimers were considered WT. **g** The frequency of latency determined by limiting dilution nested PCR of intact splenocytes for the viral genome at d42 post-challenge of mice (*N* = 3) with one or two boosts post-prime.

Since vaccination with RDV-50.stop promoted cellular and humoral immune responses directed to MHV68, we sought to determine if the vaccine response was protective in vivo. C57BL/6 mice were vaccinated with RDV-50.stop as described above or using PBS as a sham control. 14-17 days after the final dose, mice were challenged intranasally (IN) with 1000 PFU of WT MHV68 (Fig. 2a). Plaque assays performed on homogenized lung tissue day 7 (d7) post-challenge revealed significantly lower viral titers in lungs of vaccinated animals, with mice that received either one or two boosts exhibiting a three-log reduction in viral titers compared to sham-vaccinated controls (Fig. 2b). These data demonstrate that vaccination with RDV-50.stop potently inhibits WT virus replication at a mucosal barrier.

Since latent GHV infection is associated with numerous malignancies, a major goal in the development of GHV vaccines is to reduce latent infection and reactivation. We therefore sought to determine the impact of RDV vaccination on MHV68 latency. MHV68 reaches the splenic lymphoid tissue after viral transit through the lymph node and hematogenous dissemination^54^. An increase in spleen weight reflects MHV68 colonization of the spleen that occurs in an infectious mononucleosis-like syndrome during MHV68 latency establishment^55^. Infection of sham-vaccinated animals with WT MHV68 resulted in a mean three-fold increase in spleen weight relative to naive mice d16 post-challenge (Fig. 2c). While vaccination alone did not cause an increase in spleen weight, vaccination with RDV-50.stop prior to WT virus challenge prevented infection-related splenomegaly. In limiting-dilution PCR analyses to measure the frequency of splenocytes that harbor MHV68 genomes, we found that vaccination prior to the WT virus challenge resulted in a profound reduction in latently infected cells from approximately 1 in 38 splenocytes containing viral genomes in sham-vaccinated animals to 1 in 2380 after vaccination (Fig. 2d).

Latent GHVs undergo periodic reactivation that may facilitate virus persistence and transmission and are also thought to contribute to virus-driven oncogenesis and inflammatory diseases^6,56,57^. Approximately 10% of genome-positive splenocytes undergo spontaneous reactivation upon explant at 16 dpi^36^. To determine the effect of vaccination with RDV-50.stop on MHV68 reactivation from latency, we performed serial dilutions of explanted splenocytes from vaccinated and sham-vaccinated animals on an indicator monolayer and evaluated cytopathic effects at d16 post-challenge. While splenocytes from sham-vaccinated mice exhibited an ex vivo reactivation frequency of approximately 1 in 2000 splenocytes, reactivation frequencies were below the limit of detection after challenging vaccinated animals with WT MHV68 (Fig. 2e). These data suggest that immunity induced by vaccination with RDV-50.stop inhibits latency establishment and the reactivation that ensues upon explant.

RTA expression is not essential to establish latent infection of B cells in vivo^58^. LANA, the viral episomal maintenance protein encoded by *ORF73*, and other viral latency-associated genes were expressed in the absence of RTA (Fig. 1a), which led us to hypothesize that RDV-50.stop was competent for latency establishment in the spleen. To differentiate the RDV-50.stop vaccine virus from WT challenge virus, we performed semi-quantitative PCR analyses using RDV-50.stop specific primer pairs to determine the relative percentage of vaccine-derived virus present in spleens after WT challenge. As expected, each group of animals infected with a single strain of virus (either RDV-50.stop or WT MHV68) was positive only for the respective virus they received (Fig. 2f). In contrast, DNA from spleens of vaccinated mice that were challenged with WT virus contained a mixture of viral genomes. In a standardized quantity of spleen DNA, only 13.9% of viral DNA amplified was from the vaccine strain. When we extended this analysis to d42 post-challenge, we observed overlapping frequencies of MHV68 genome-positive splenocytes in sham-vaccinated and vaccinated animals (Fig. 2g). Taken together, lytic replication and latency establishment by WT MHV68 is potently restricted by prime-boost vaccination with RDV-50.stop, but it does not elicit sterilizing immunity against persistence of WT virus latency.

### Virus-specific immune responses correlate with protection from WT challenge

**Fig. 3:**
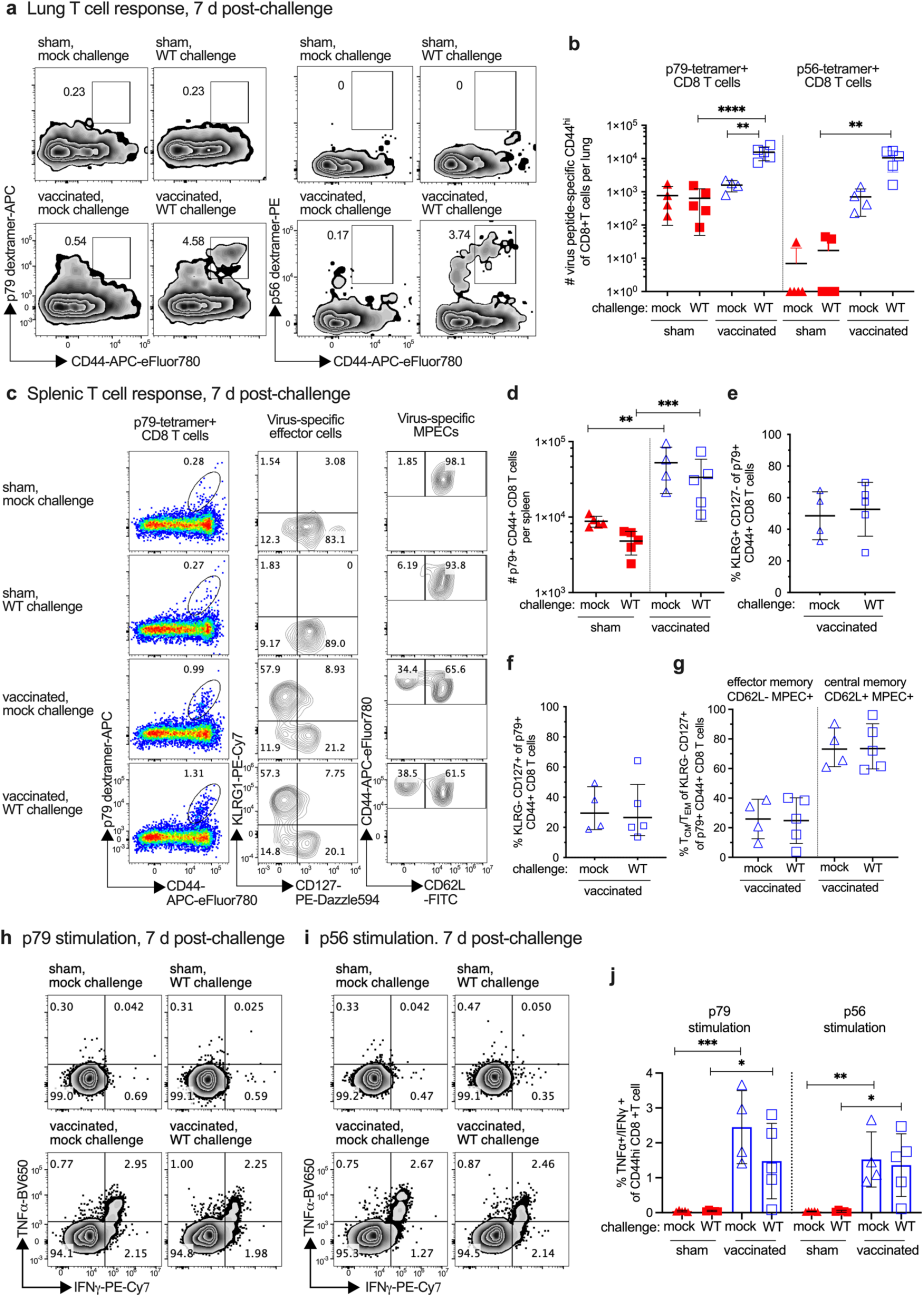
Evaluation of T cell response to MHV68 in the lungs and spleens of vaccinated mice at seven days post-challenge with WT virus. C57BL/6 mice were either sham-vaccinated or vaccinated twice (prime+boost) with 1 × 10^6^ PFU RDV-50.stop followed by mock challenge or IN challenge with 1 × 10^3^ PFU WT MHV68 at d15 post-boost and analyzed d7 post-challenge. **a** Flow cytometric gating strategy to determine the frequency of CD44^hi^ CD8 T cells in the lungs that were reactive with viral p79 or p56 epitopes. **b** Total p79- or p56-tetramer+ CD8 T cells per lung of individual mice with the indicated vaccination and challenge regimen. **c** Left column, flow cytometric gating strategy to determine the frequency of CD44^hi^ CD8 T cells in the spleens that were reactive with viral p79. Middle column, p79-tetramer+ CD8 T cells were further analyzed for markers of short-lived effector cell (SLEC, KLRG^+^CD127^−^) and memory precursor effector cell subsets (MPEC, KLRG^−^CD127^+^). Right column, MPECs were further delineated into CD62L^−^ effector and CD62L^+^ central MPECs. **d** Total p79-tetramer+ CD8 T cells per spleen of individual mice with the indicated vaccination and challenge regimen. The percentage of p79-reactive CD8 T cells that were SLECs **e**, MPECs **f**, and effector vs memory MPECs **g** were enumerated based on the gating strategy for surface markers in **c**. Intracellular cytokine levels of effector cytokines TNFα and IFNγ were examined 6 h after stimulation with **h** p79 and **i** p56 peptides. **j** Percentage of CD44^hi^ CD8 T cells producing both TNFα and IFNγ. For each graph, symbols represent individual mice, (*N* = 4–5); bars and whiskers are mean +/− SD. *, *p* < 0.05; **, *p* < 0.05; ***, *p* < 0.001; ****, *p* < 0.0001 in Sidak’s multiple comparisons test of one-way ANOVA between the indicated groups.

Identification of immune correlates of protection is an important goal of developing an effective vaccine for GHVs and other viruses. We therefore determined the effect that RDV-50.stop vaccination had on immune activation following WT challenge. In analyses of T cell responses during the acute phase, CD8 T cells specific for the MHV68 p79 and p56 immunodominant epitopes were detected in the lungs, a primary site of infection after IN inoculation, of vaccinated mice d7 post-challenge (Fig. 3a, b). In spleens, CD8 T cells specific for p79 (Fig. 3c, d) and p56 (Supplementary Fig. 2a) were abundant in vaccinated mice, with or without WT challenge. Further immunophenotyping revealed that over 50% of p79-specific (Fig. 3c, d) and 30% of p56-specific (Supplementary Fig. 2b) CD44^+^ CD8 T cells in vaccinated mice were KLRG1^+^CD127^−^, consistent with a short-lived effector cell (SLEC) phenotype (Fig. 3e). CD62L analysis of KLRG1^−^CD127^+^ memory precursor effector CD8 T cells (MPEC) identified a similar proportion of CD62L^−^ effector memory MPEC compared to CD62L^+^ central memory MPEC in vaccinated mice regardless of wild-type challenge (Fig. 3c, f, g and Supplementary Fig. 2c, d). Stimulation of splenic CD8 T cells with p79 (Fig. 3h) or p56 (Fig. 3i) peptides led to increased production of antiviral effector cytokines TNFα and IFNγ in vaccinated mice (Fig. 3j), demonstrating that antigen-specific cells were present and responsive. Virus-specific neutralizing antibodies were also detected in RDV-50.stop vaccinated mice d7 post-challenge (Supplementary Fig. 2e, f). Taken together, prime-boost vaccination with RDV-50.stop elicited virus-specific CD8 T cells that are poised to respond to WT challenge during the acute phase of infection. Thus, neutralizing antibody and virus-specific CD8 T^eff^ responses correlate with the strong protection against WT replication and latency-dependent reactivation that is afforded to vaccinated mice (Fig. 2).

**Fig. 4:**
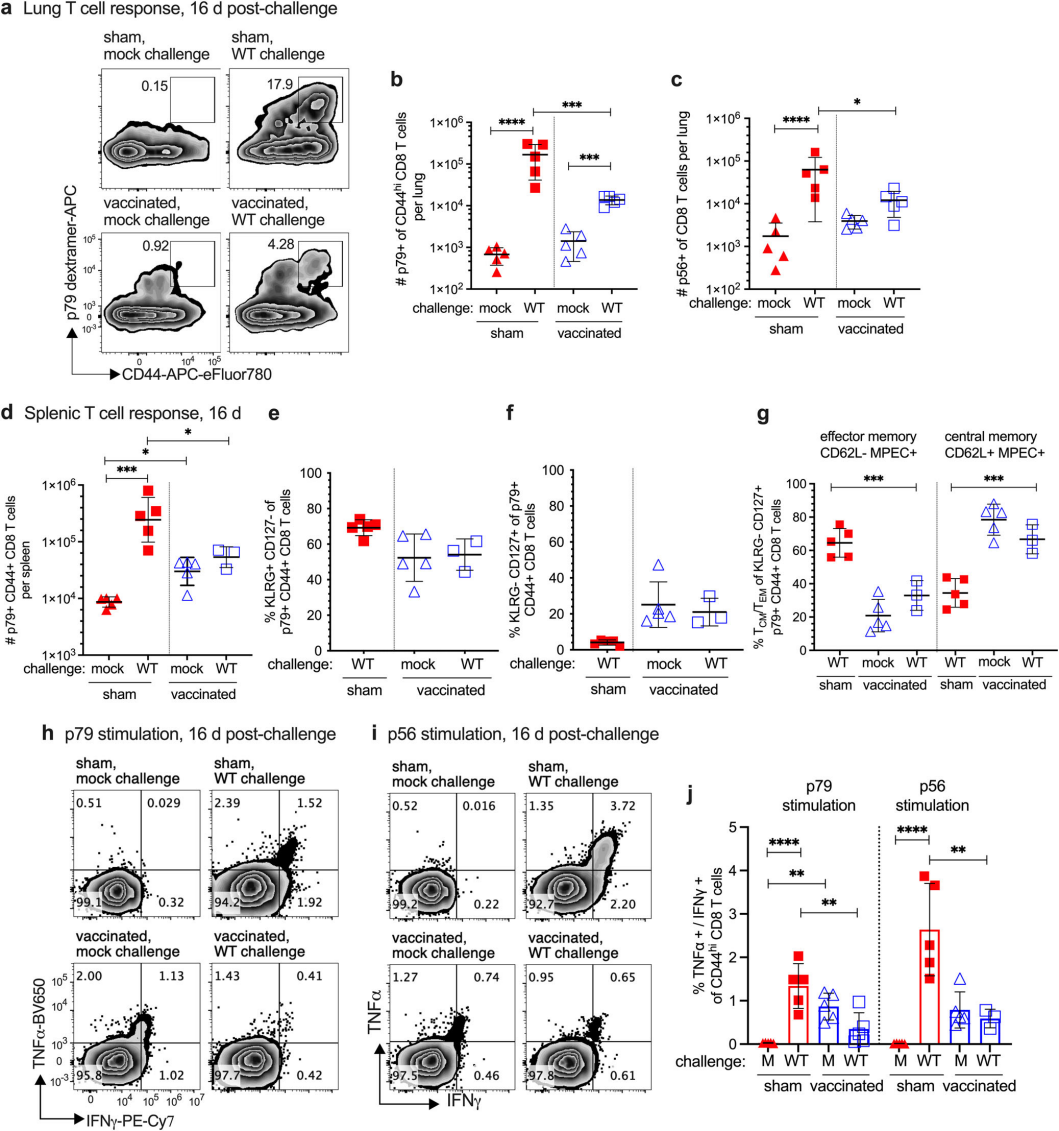
Evaluation of T cell response to MHV68 in the lungs and spleens of vaccinated mice at sixteen days post-challenge with WT virus. C57BL/6 mice were either sham-vaccinated or vaccinated twice (prime+boost) with 1×10^6^ PFU RDV-50.stop followed by mock challenge or IN challenge with 1×10^3^ PFU WT MHV68 at d15 post-boost. **a** Flow cytometric gating strategy to determine the frequency of CD44^hi^ CD8 T cells in the lungs of individual mice with the indicated vaccination and challenge regimen that were reactive with viral p79 at d16 post-challenge. **b**, **c** Total p79- or p56-tetramer+ CD44^hi^ CD8 T cells per lung of individual mice after initial prime and sequential boost. **d** Total p79-tetramer+ CD44^hi^ CD8 T cells per spleen of individual mice after initial prime and sequential boosts. Percentage of p79-tetramer+ CD44^hi^ CD8 T cells with markers of **e** short-lived effector cell (SLEC, KLRG^+^CD127^−^) and **f** memory precursor effector cell subsets (MPEC, KLRG^−^CD127^+^). **g** MPECs were further delineated into CD62L^−^ effector and CD62L^+^ central MPECs for p79-tetramer+ CD8 T cells. Intracellular cytokine levels of effector cytokines TNFα and IFNγ were examined 6 h after stimulation with **h** p79 and **i** p56 peptides. **j** Percentage of CD44^hi^ CD8 T cells producing both TNFα and IFNγ in response to viral peptide stimulation. For each graph, symbols represent individual mice, (*N* = 3–5); bars and whiskers are mean +/− SD. *, *p* < 0.05; **, *p* < 0.05; ***, *p* < 0.001; ****, *p* < 0.0001 in Sidak’s multiple comparisons test of one-way ANOVA between the indicated groups.

The CD8 T cell response was next compared between the sham-vaccinated and RDV-50.stop vaccinated mice at d16 post-challenge, a timepoint of peak virus latency in the early chronic phase of WT infection. A potent virus-specific CD8 T cell response was observed at d16 post-challenge in the lungs of sham-vaccinated mice that surpassed the levels detected in the vaccinated mice upon challenge (Fig. 4a–c), consistent with the typical kinetics of the CD8 T cell response that arrests WT virus replication in the lung by 9-12 dpi. A similar observation was made for the spleen. Virus-specific CD8 T cells in the spleens of sham-vaccinated animals exceeded those in the vaccinated mice, and these T cells had an increased SLEC and CD62L^−^ effector MPEC phenotype d16 post-challenge, for both p79 (Fig. 4d–g) and p56 (Supplementary Fig. 3a–d) epitopes. Production of antiviral TNFα and IFNγ effector cytokines correlated with this increased CD8 T^eff^ phenotype in spleens of WT infected mice that were not vaccinated compared to their RDV-50.stop vaccinated counterparts (Fig. 4h–j). In summary, vaccinated mice challenged with WT virus exhibited a reduced CD8 T cell response compared to their non-vaccinated counterparts at d16 post-challenge. This observation is consistent with a strong pre-existing immune response in the lungs and spleen afforded by RDV-50.stop vaccination (Fig. 3) that limits viral replication (Fig. 2). In contrast, sham-vaccinated mice are unable to control initial viral replication and elicit a larger CD8 T cell response at d16 post-challenge.

### RDV-50.stop demonstrates greater efficacy than the heat-inactivated vaccine against the wild-type MHV68 challenge

**Fig. 5:**
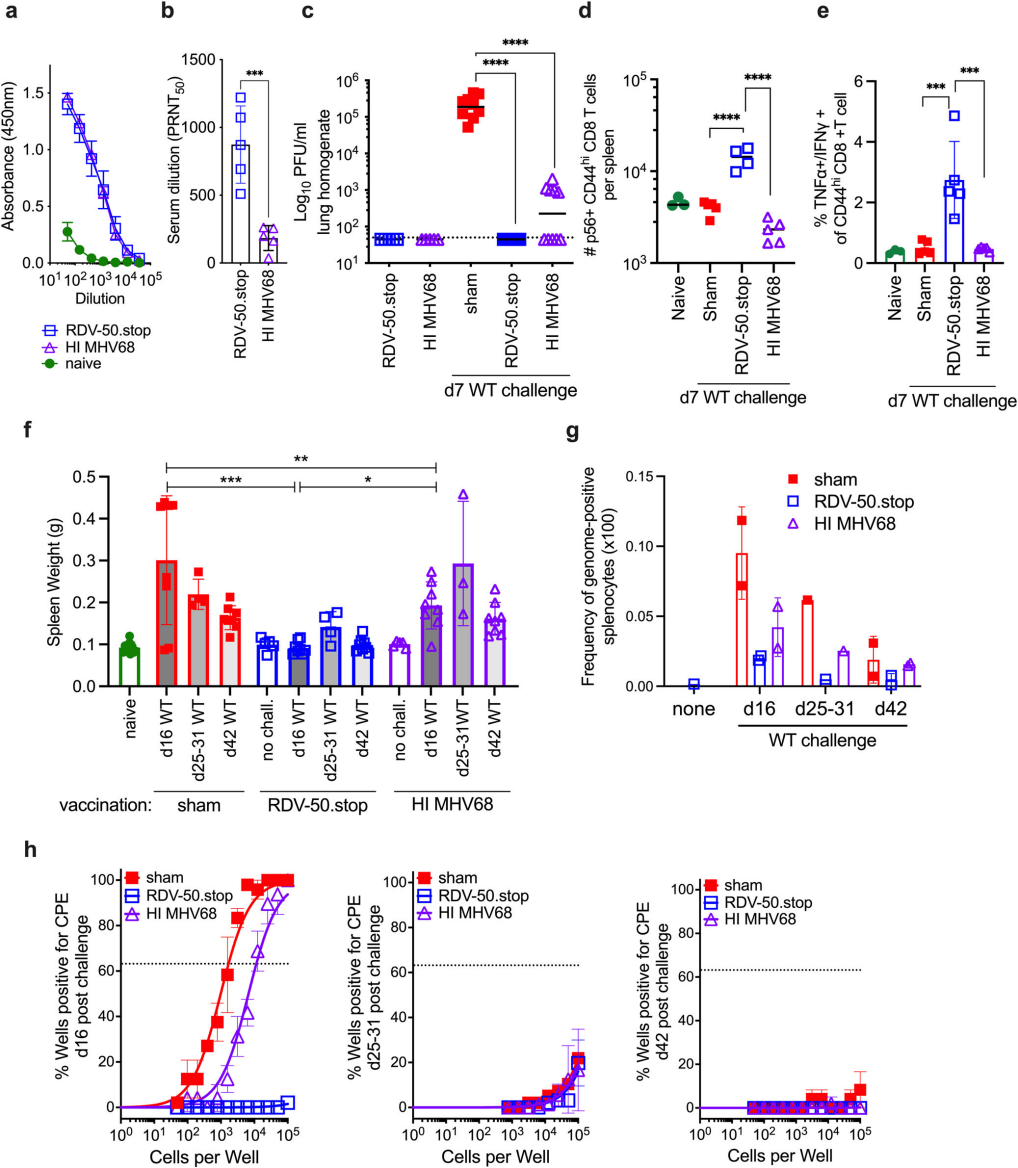
RDV-50.stop demonstrates greater efficacy when compared to a heat-inactivated vaccine against wild-type MHV68 challenge. C57BL/6 mice were either sham-vaccinated or vaccinated (prime+boost) with 1 × 10^6^ PFU RDV-50.stop or heat-inactivated (HI) WT MHV68. **a** Virus-specific IgG from RDV-50.stop or HI vaccinated mice at d31 post-boost measured by ELISA. **b** Virus neutralization in serum at d31 post-boost determined by a plaque reduction assay. The plaque reduction neutralization titer 50 (PRNT_50_) value is the dilution of serum to reach 50% neutralization of plaques. For **a-b**, symbols denote individual mice (*N* = 5); bars and whiskers represent mean +/− SD. ***, *p* < 0.001 in unpaired two-tailed *t*-test. **c-f** Following prime+boost vaccination, mice were challenged IN with 1 × 10^3^ PFU WT MHV68 at d14 post-boost. **c** Acute replication at d7 post-challenge determined by measuring infectious particles per ml lung homogenate. Dotted line indicates limit of detection at 50 PFU/ml. **d** Total p56-tetramer+ CD44^hi^ CD8 T cells per spleen of individual mice at d7 post-challenge. **e** Percentag**e** of CD44^hi^ CD8 T cells producing both TNFα and IFNγ in response to viral p56 and p79 peptide dual stimulation at d7 post-challenge. For **c**–**e**, symbols denote individual mice (*N* = 5–10); bars and whiskers represent mean +/− SD. ***, *p* < 0.001; ****, p < 0.0001; in Dunnett’s **c** or Sidak’s **d**, **e** multiple comparisons test of one-way ANOVA between the indicated groups. **f** Splenomegaly determined by spleen weights at d16, d25-31 and d42 post-challenge, respectively. Symbols denote individual mice (*N* = 3–); bars and whiskers represent mean +/− SD. *, *p* < 0.05; **, p < 0.001; ***, *p* < 0.005; in Sidak’s multiple comparisons test of one-way ANOVA between the sham, RDV-50.stop and HI MHV68 vaccinated groups. **g** The frequency of genome-positive splenocytes determined by limiting dilution nested PCR of intact splenocytes at d16, d25-31 and d42 post-challenge. **h** The frequency of explant reactivation determined by limiting dilution coculture of intact viable splenocytes on a monolayer of primary MEFs d16, d25-31 and d42 days post-challenge. Disrupted splenocytes plated in parallel did not reveal preformed infectious virus in the vaccinated animals.

To further examine the efficacy of RDV-50.stop, we compared vaccination with RDV-50.stop to a heat-inactivated (HI) preparation of WT MHV68 vaccination approach, a commonly used strategy that was previously reported for MHV68^40^. First, C57BL/6 mice were prime-boost vaccinated with 10^6^ PFU of either RDV-50.stop or HI WT MHV68, in the absence of WT challenge, and analyzed at d31 post-boost. Total MHV68-specific IgG elicited by HI vaccination mice was comparable to RDV-50.stop (Fig. 5a), but RDV-50.stop elicited significantly higher levels of virus-neutralizing antibodies compared to HI vaccinated mice (Fig. 5b). Unlike RDV-50.stop, HI MHV68 vaccination did not elicit CD8 T cell responses to immunodominant epitopes from ORF6 and ORF61 in the lungs (Supplementary Fig. 4a, b) and spleen (Supplementary Fig. 4c-e). No infectious virus was detected upon vaccination with RDV-50.stop or the HI in the lungs (Fig. 5c) or spleen (Supplementary Fig. 4f) confirming the vaccine preparations did not contain or produce infectious particles.

When challenged intranasally with 1000 PFU of WT MHV68 at d14 post-boost, HI vaccination was only partially protective against acute replication at d7 post-challenge, while RDV-50.stop again completely blocked viral replication (Fig. 5c). HI MHV68 vaccination did not elicit CD8 T cell responses to p56 (Fig. 5d) or p79 (Supplementary Fig. 4g). CD8 T cells from mice vaccinated with HI MHV68 also failed to produce antiviral cytokines TNFα and IFNγ upon dual stimulation with p56 and p79 peptides (Fig. 5e), suggesting inferior virus-specific T cell control compared to RDV-50.stop vaccination. By d16 post-challenge, mice vaccinated with HI MHV68 exhibited no significant protection against splenomegaly (Fig. 5f) and only partial protection against early establishment of splenic latency (Fig. 5g). Reflecting the reduction in latency establishment after HI MHV68 vaccination, reactivation from explanted splenocytes was reduced approximately seven-fold, but the RDV-50.stop vaccination again resulted in levels below the limit of detection upon WT challenge (Fig. 5h). The analysis of splenic latency at intermediate (d25-31) and late (d42) timepoints after WT viral challenge revealed that long-term latency was not prevented by HI MHV68 vaccination as found for RDV-50.stop vaccinated mice (Fig. 5g). Reactivation was barely detectable in splenocytes from all groups upon explant at intermediate and late timepoints and no preformed infectious virus was detected in vaccinated mice (Fig. 5h). Overall, these data indicate that RDV-50.stop vaccination is superior to the commonly used heat-inactivation strategy of vaccination in the MHV68 model, especially in eliciting T cell-specific immunity against the virus.

### Durable protection with RDV-50.stop vaccination

**Fig. 6:**
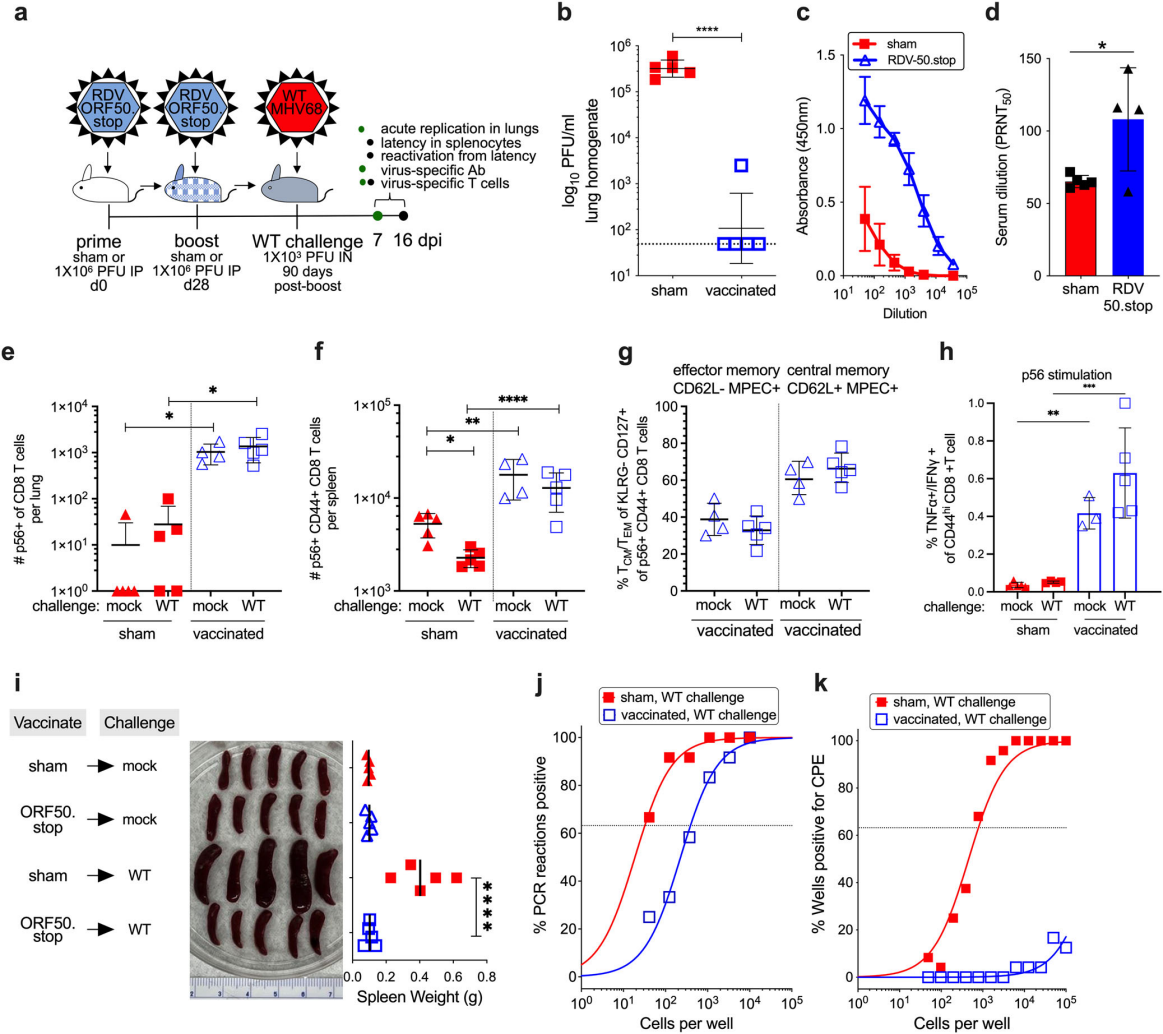
Vaccination with RDV-50.stop leads to durable protection against wild-type MHV68 challenge. **a** C57BL/6 mice were either sham-vaccinated or vaccinated twice (prime+boost) with 1 × 10^6^ PFU RDV-50.stop followed by IN challenge with 1 × 10^3^ PFU WT MHV68 at 90 days post-boost. **b** Acute replication at d7 post-challenge determined by measuring infectious particles per ml lung homogenate. Symbols denote individual mice (*N* = 5); bars and whiskers represent mean +/− SD. ****, *p* < 0.0001 in one-tailed unpaired t test of log-transformed values. Dotted line indicates limit of detection at 50 PFU/ml. **c** Virus-spe**c**ific IgG from sham or RDV-50.stop vaccinated mice at d7 post-challenge measured by ELISA. Symbols denote individual mice (*N* = 4); bars and whiskers represent mean +/− SD. **d** Virus neutralization in serum as determined by a plaque reduction assay. The plaque reduction neutralization titer 50 (PRNT_50_) value is the dilution of serum to reach 50% neutralization of plaques. Symbols denote individual mice (*N* = 4–5); bars and whiskers represent mean +/− SD. *, p < 0.05 in two-tailed unpaired *t*-test. **e**, **f** Total p56-tetramer+ CD44^hi^ CD8 T cells per lung **e** or spleen **f** of individual mice. **g** MPECs were delineated into CD62L^−^ effector and CD62L^+^ central MPECs for p56^−^tetramer+ CD44^hi^ CD8 T cells. **h** Percentage of CD44^hi^ CD8 T cells producing both TNFα and IFNγ in response to viral p56 peptide stimulation. For **d**–**h**, symbols represent individual mice, (*N* = 3-5); bars and whiskers are mean +/− SD. *, *p* < 0.05; **, *p* < 0.05; ***, *p* < 0.001; ****, *p* < 0.0001 in Dunn’s **e** or Sidak’s **f,h** multiple comparisons test of one-way ANOVA betwe**e**n the indicated groups. **i** Splenomegaly visualized for whole spleens and quantitated by spleen weights. Symbols represent individual mice, (*N* = 5); bars and whiskers represent mean +/− SD. ****, *p* < 0.0001 in Tukey’s test of one-way ANOVA. **j** The frequency of latency determined by limiting dilution nested PCR of intact splenocytes for the viral genome. **k** The frequency of explant reactivation determined by limiting dilution coculture of intact viable splenocytes on a monolayer of primary MEFs. Disrupted splenocytes plated in parallel did not detect preformed infectious virus in the vaccinated animals. Error bars represent SEM. Symbols denote the average of one experiment with five mice per experiment.

To test the durability of RDV-50.stop vaccine-induced immunity, we evaluated the impact of vaccination on WT virus challenge 90 days after completion of the prime-boost regimen (Fig. 6a). In vaccinated mice, viral titers in lungs were undetectable by plaque assay in four of five animals at d7 post-challenge (Fig. 6b). This correlated with detection of virus-specific, neutralizing antibodies (Fig. 6c, d) and the presence of virus-specific CD8 T cells in the lungs and spleens of vaccinated mice independent of challenge (Fig. 6e, f). Moreover, CD8 T cells recognized by the p56 MHCI tetramer in spleens of vaccinated mice predominantly bore markers of central memory MPEC cells (Fig. 6g) and produced antiviral cytokines TNFα and IFNγ upon p56 peptide stimulation (Fig. 6h) at d7 post-challenge.

With regard to durability of protection against WT MHV68 latency establishment, the mean splenomegaly was reduced by four-fold in RDV-50.stop vaccinated mice compared to sham-vaccinated mice on d16 post-challenge (Fig. 6i). Congruent with decreased splenomegaly, vaccination also resulted in a 10-fold reduction in the frequency of viral genome-positive cells in comparison to non-vaccinated controls (Fig. 6j), and virus reactivation was well below the threshold of accurate quantitation, reduced by at least three orders of magnitude (Fig. 6k). At d16 post-challenge, the number of virus-reactive CD8 T cells in lungs (Supplementary Fig. 5a-b) and spleens (Supplementary Fig. 5c, g) were lower in vaccinated compared to sham-vaccinated animals. The virus-specific CD8 T cells had a decreased SLEC profile and production of antiviral cytokines (Supplementary Fig. 5d–f, h–k) were reduced in RDV-50.stop vaccinated mice, with or without challenge, compared to sham-vaccinated mice. This suggests that either the anamnestic response to re-infection in vaccinated animals requires less activation and expansion to be effective or that local immune control in the lung reduces the general need for immune expansion to control MHV68 infection in the spleen. Overall, these data indicate that the protective immune response generated by vaccination with RDV-50.stop is durable and protective up to 3 months post-boost.

### Vaccination with RDV-50.stop protects mice lacking type I interferon responses from severe disease

GHV infection can lead to a diverse array of cancers, inflammatory disease, and even death in hosts with weakened adaptive and innate immune systems^59^. Type I interferon signaling is required to control lytic replication and reactivation of MHV68 from latency^60,61^. While adaptive immune responses remain functional, mice lacking the type 1 interferon receptor (*Ifnar1*^*−/−*^) are incapable of responding to interferon-ɑ/β, leading to an increase in susceptibility to severe disease, acute viral replication, and mortality when infected with MHV68^62^. To determine if RDV-50.stop vaccination was protective in a host that is highly susceptible to severe disease, we vaccinated mice lacking *Ifnar1*.

**Fig. 7:**
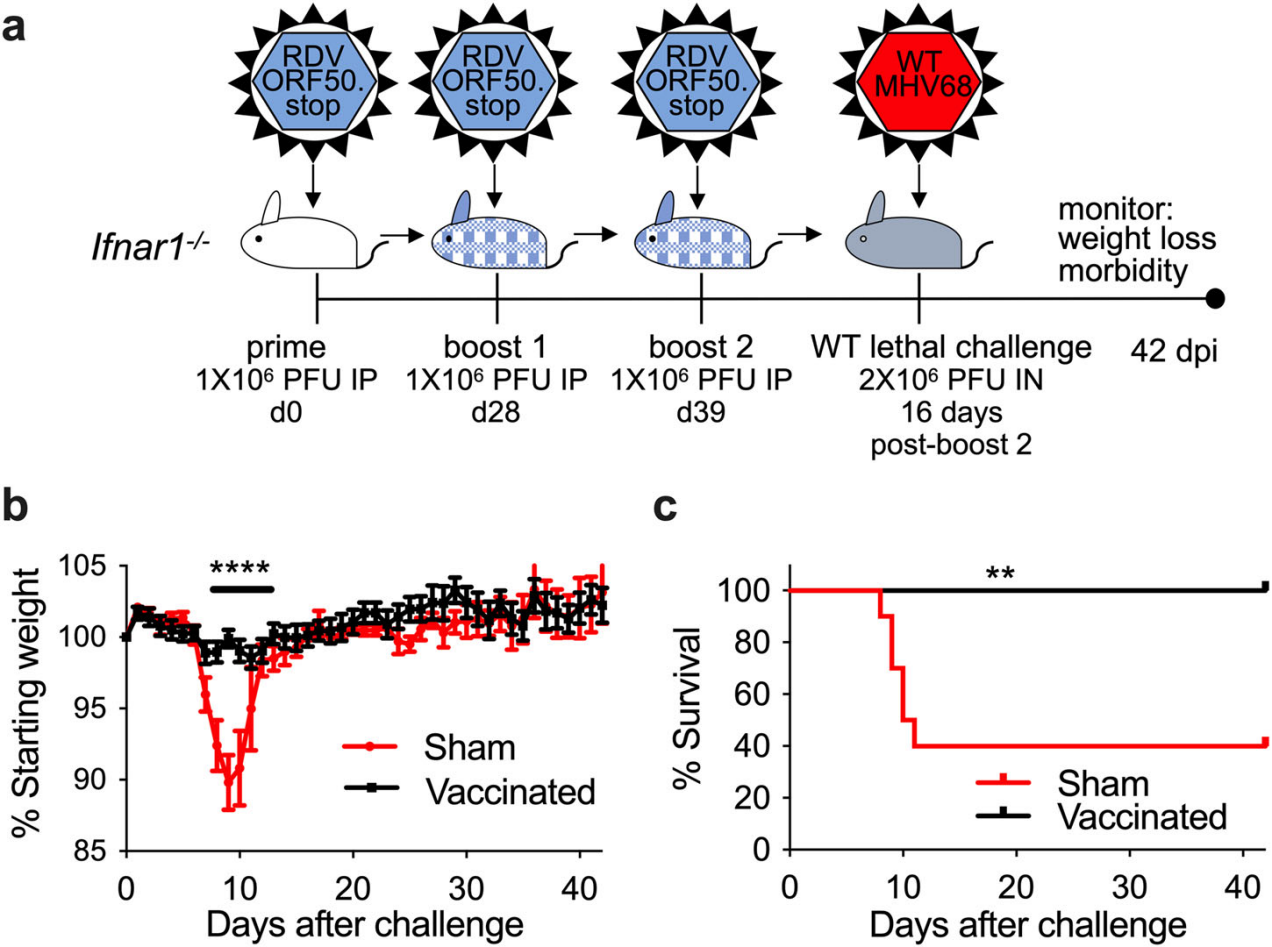
Vaccination with RDV-50.stop protects mice susceptible to severe disease from weight-loss and disease. **a** Groups of 10 C57BL/6 *Ifnar1*^*−/−*^ mice were either sham-vaccinated or vaccinated with 3 doses of 1 × 10^6^ PFU RDV-50.stop MHV68 and challenged with a lethal dose of 2 × 10^6^ PFU WT MHV68. Sham-vaccinated and vaccinated mice were weighed daily for 42 days to measure disease progression. **b** Daily weights were averaged to determine the protective effect of the vaccinated group. **c** Survival curve for sham-vaccinated and vaccinated *Ifnar1*^*−/−*^ mice. Symbols represent individual mice; error bars represent SEM. ****, *p* < 0.0001 in a two-tailed t test; **, *p* < 0.01 in a logrank test.

*Ifnar1*^*−/−*^ mice were sham-vaccinated or vaccinated according to the previously established timeline (Fig. 7a). Similar to WT C57BL/6 mice, vaccination of *Ifnar1*^*−/−*^ mice with RDV-50.stop resulted in an increase in virus-specific, neutralizing antibody production (Supplementary Fig. 6). Two weeks after completing the vaccination regimen, *Ifnar1*^*−/−*^ mice were challenged intranasally with 2x10^6^ PFU of WT MHV68, a dose previously determined to be lethal in *Ifnar1*^*−/−*^ mice on a C57BL/6 background^63^, and monitored for signs of disease for 6 weeks after challenge. Mice were weighed daily for 42 days and assessed for signs of distress. Sham-vaccinated animals began losing weight on d5 post-challenge, with 90% of animals losing greater than 10% body weight by d10 post-challenge (Fig. 7b and Supplementary Fig. 7a). By d11 post-challenge, 6 out of 10 sham-vaccinated *Ifnar1*^*-/*-^ reached humane endpoint criteria (Fig. 7c). In contrast, none of the vaccinated *Ifnar1*^*−/−*^ mice showed signs of disease; weights remained stable, and animals remained mobile and active throughout the course of the study (Fig. 7b, c and Supplementary Fig. 7b).

Consistent with the effects of vaccination observed for WT mice, vaccination with RDV-50.stop MHV68 resulted in a significant decrease in the frequency of splenocytes harboring viral genomes. For surviving sham-vaccinated mice, approximately 1 in 100 splenocytes were viral-genome positive, compared to 1 in 3500 splenocytes in vaccinated animals (Supplementary Fig. 8a). When the source of viral genomes was evaluated by genotyping PCR, 14% of the virus present was vaccine-derived (Supplementary Fig. 8b), which further indicates that vaccination with RDV-50.stop is clearly protective against severe disease, but it does not generate sterilizing immunity against latent colonization by a WT virus.

## Discussion

In this study, we demonstrate the potential for a replication-deficient gammaherpesvirus to elicit a potent immune response and protect from WT replication, latency establishment, and disease in vivo. RDV-50.stop functions as a hybrid vaccine platform, capitalizing on the complex antigenicity of the virus particle itself combined with its ability to infect antigen-presenting cells to permit antigen presentation of virion-associated proteins and the limited genes expressed from the delivered viral genome. The absence of wild-type revertants using our production strategy, the benefit of eliciting responses to structural and non-structural viral proteins, and the implementation of a boost are novel aspects of our vaccine approach.

Cellular immune responses are understudied in the context of human GHV vaccinology. MuMT mice, which lack the capacity to form B cells, are capable of controlling acute MHV68 infection due to the cellular response to infection^64^. Mice lacking either CD4 or CD8 T cells are less capable of controlling acute viral replication and reactivation from latency, resulting in increased susceptibility to severe disease and lymphoproliferation^47,65–69^. Here, humoral and cellular-mediated immune responses of vaccinated mice were monitored prior to and upon the WT challenge. The neutralizing titer of serum and effector T cells specific to known immunodominant epitopes from lytic proteins increased following prime-boost dosing of WT C57BL/6 mice with RDV-50.stop. Fc-dependent antibody-mediated cytotoxicity and phagocytosis should be evaluated. Clearance of acute MHV68 replication in the lungs requires CD8, but not CD4 T cells^66,70^. In the lungs and spleens of RDV-50.stop vaccinated mice, there was an effector CD8 T cell population specific for the major lytic viral antigens p79, an epitope present within the viral large ribonucleotide reductase subunit encoded by *ORF61*, and p56 an epitope derived from the viral single-strand DNA binding protein encoded by *ORF6*. Transcripts from these genomic regions were detected in fibroblasts infected with RDV-50.stop, suggesting that these are RTA-independent genes expressed in abortively infected cells or transiently expressed during latency. Following the WT MHV68 challenge, virus-specific CD8 T cells were further elevated by d7, suggesting that RDV-50.stop vaccination primed the T cell response for the challenge. Viral titers from lung homogenates collected on day 7 were significantly reduced by three orders of magnitude in vaccinated mice, often to levels below the limit of detection. It is interesting to speculate that the T-cell response that occurred after the WT virus challenge in vaccinated animals reflects an increase in the local capacity to control infection at the mucosal barrier. Future studies may evaluate whether the increase in virus-specific T cells is due to recruitment or expansion. Taken together, pre-existing humoral and cellular immunity produced by prime-boost immunization with an RDV controls acute replication. Moreover, this response was durable, as protection was maintained up to 3 months post-vaccination.

At d16 post-challenge with WT MHV68 in unvaccinated mice, acute infection has been cleared and latency is established. Indeed virus-specific T-cell responses in sham-vaccinated mice surpassed those of vaccinated animals after the WT virus challenge presumably due to the higher viral load present in the absence of immune control afforded by the vaccine. RDV-50.stop led to a 10-fold reduction in genome-positive splenocytes compared to sham-vaccinated control mice. Likewise, reactivation from explanted cells was nearly abolished in the vaccinated animals. Latency establishment and reactivation were analyzed over a six-week time course. The low levels of latency that were established at early times, in spite of vaccination, were maintained through the later time points. There was no evidence of pre-formed infectious WT virus and the levels of WT virus reactivation from vaccinated mice upon explant were below the limit of detection at all time points. WT MHV68 gains access to immunoglobulin class-switched memory B cells as a latency reservoir that is stable through late, chronic infection timepoints^71,72^. We previously demonstrated that viral replication in B cells is not required for MHV68 latency establishment and maintenance^58,73,74^, supporting a model wherein the B cell latency reservoir is maintained in the absence of reactivation. Multiple mechanisms might contribute to the appearance of stable levels of latency over time, even after vaccination: a) latency may be established in a stable reservoir as early as d16 post-challenge; b) proliferative expansion may occur as the gammaherpesvirus latency program expresses numerous viral factors that promote B cell proliferation; and c) reactivation from latency may be occurring at very low levels in vivo, beyond our ability to accurately quantify. Importantly, any of these scenarios are occurring despite a virus-specific immune response that controls replication and protects *Ifnar1*^*−/−*^ mice from lethality. We believe findings from this study highlight a major challenge for prophylactic vaccine efforts against the gammaherpesviruses.

When compared with a heat-inactivated WT MHV68 vaccine, RDV-50.stop demonstrated an overall higher protective efficacy against WT MHV68 challenge. Vaccination with HI WT MHV68 led to partial protection against acute replication and splenic latency, consistent with a previous report^40^. However, the inactivated vaccine did not elicit CD8 T cell responses to immunodominant epitopes of MHV68 in C57BL/6 mice, suggesting inferior virus-specific T cell control. Notably, the epitopes are derived from the nonstructural ORF6 viral DNA replication protein and the ORF61 ribonucleotide reductase L subunit that is expressed upon abortive infection with RDV-50.stop. This demonstrates a major advantage of an infectious viral vaccine that expresses nonstructural antigens in a single round of non-productive infection. While the HI MHV68 vaccine was less effective, the partial protection in lungs during acute viral replication did correlate with detection of virus-neutralizing antibody in serum. It is also possible that T cell responses were present against other viral antigens that remain to be identified. Importantly, these findings support the utility of the MHV68 system to define how standard and novel vaccination approaches differentially influence the development of immune protection against gammaherpesvirus replication, latency, and virus-driven disease.

Within immunocompromised individuals, GHV infection can lead to the onset of a plethora of lymphoproliferative disorders^75^. Previously studied latency-defective, yet replication-competent viruses, were found to provide protection from WT MHV68 challenge^39,40^, but a replication-competent virus may pose a risk for hosts lacking innate immune control. Because of this, we wanted to determine if RDVs were both safe and effective in reducing the incidence of disease in a model of innate immune deficiency. To eliminate the risk of WT contamination and reversion, we grew RDV-50.stop on cell lines expressing a codon-shuffled *ORF50* construct, limiting sequence homology to inhibit recombination while still providing the WT protein required for replication^46^. Indeed, we previously reported that 1 × 10^6^ infectious RDV-50.stop particles produced on the codon-shuffled producer cells were not lethal in SCID mice, whereas mortality occurs with as few as 10 PFU of WT virus^46^. To determine if RDV vaccination was protective in cases where a host is susceptible to severe disease, we vaccinated mice lacking the type I interferon receptor common alpha subunit*. Ifnar1*^*−/−*^ mice experience acute disease leading to mortality following MHV68 infection^62,63^. RDV prime-boost vaccination of *Ifnar1*^*−/−*^ mice increased virus-specific antibody titers with neutralizing capacity, similar to immune competent C57BL/6 mice. RDV vaccination led to a striking, complete protection from WT MHV68 challenge, as all vaccinated mice maintained consistent body weights and displayed no outward signs of distress. Of those sham-vaccinated *Ifnar1*^*−/−*^ mice that survived to the endpoint of the experiment, latent infection was ten-fold greater than vaccinated controls. RDV vaccination is therefore effective at inducing immune responses that correlate with protection from acute infection and disease in hosts susceptible to severe disease.

While our data demonstrate the protective potential of an RDV-based vaccine strategy, there are a few caveats with the RDV vaccine platform. While we noted lower overall levels of latent infection following WT GHV challenge, differential PCR demonstrates that RDV vaccination did not yield sterilizing immunity, as WT MHV68 comprises the bulk of the latent reservoir in vaccinated animals. It is worth noting that the phase 2 clinical trial of the EBV gp350 subunit vaccine reduced the incidence of infectious mononucleosis even though EBV infection was not blocked^32,33^. Given the capacity of GHVs to promote proliferation of latently infected B cells in the absence of lytic replication, sterilizing immunity may be an unattainable goal. However, vaccination strategies that reduce EBV-driven IM and MS, and the numerous lymphomas and cancers caused by both EBV and KSHV is a clinically significant outcome^10,34^. Another caveat of this first-generation design is that RDV-50.stop leaves intact numerous genes that enable the establishment and maintenance of latency, notably the unique MHV68 *M* genes and *ORF73* encoding the episome maintenance protein latency-associated nuclear antigen (LANA). As such, RDV-50.stop is able to establish a chronic latent infection, albeit at lower levels relative to WT virus at d16 post-infection. Live-attenuated viruses that are unable to establish latency protect from WT MHV68 challenge^39,40^. A replication competent MHV68 vaccine virus lacking LANA, the latency protein essential for episomal maintenance, was reported to provide better protection than a replication-deficient virus that lacked both the RTA lytic transactivator and LANA^40^. Such a contrast is not surprising since only single vaccinations in the absence of adjuvant were evaluated^40^. Here, the RDV-50.stop virus was administered in the absence of an adjuvant. While boosting enhanced the humoral response to infection, the CD8 T cell response was only moderately impacted. Future studies will evaluate whether combinatorial replication and latency-defective viruses benefit from adjuvanted boosting and reach the immunogenicity required for protection. A final caveat of a replication-deficient vaccine platform is that known oncogenes for gammaherpesviruses would need to be disabled or completely deleted to prevent the potential for transformation. Live-attenuated vaccines have been instrumental in preventing multiple infections and diseases, but additional safety concerns are reversion to virulence and the potential for vaccine-driven pathology in immunocompromised individuals.

In summary, the studies described here detail the potential of a RDV-based vaccine platform to generate immune responses capable of inhibiting GHV replication and latency in immune-competent mice and protect them from death in a lethal model of disease. We observed virus-specific humoral and CD8 T cell responses upon RDV-50.stop vaccination; however, the full repertoire of the immune reactome remains to be defined. Rationally designed vaccines tested in the MHV68 model system will enable targeted depletion and transfer of specific immune components to delineate the determinants and effector mechanisms that block and control infection. Further refinement of vaccine design by delaying the block in virus replication to lytic genes of later kinetic classes and co-administration of next-generation RDVs in the context of adjuvant is expected to further expand the breadth and depth of antigen presentation and our understanding of the immune correlates of protection against gammaherpesvirus infections and their ensuing pathologies.

## Methods

### Cells and viruses

Primary C57BL/6 murine embryonic fibroblasts (MEFs), NIH 3T12 (ATCC CCL-164), BHK21 (ATCC CCL-10), Vero (ATCC CCL-81), and Vero-Cre cells were cultured in Dulbecco’s Modified Eagle Medium (DMEM) supplemented with 10% fetal bovine serum (FBS), 100 ug/ml penicillin, 100 ug/ml streptomycin, and 2 mM L-glutamine to form complete DMEM (cDMEM). Cells were maintained at constant conditions in a standard incubator at 37˚C, 5% CO_2,_ and 99% humidity. Primary MEFs at passages 2 and 3 were used for limiting dilution reactivation assays.

Viruses utilized in this study include WT MHV68 (WUMS strain) for vaccine challenge experiments in C57BL/6 mice, BAC-derived WT MHV68^76^ for comparative immune responses in C57BL/6 and vaccine challenge experiments in *Ifnar1*^*−/−*^ mice, and the replication-deficient (RDV) ORF50.stop recombinant MHV68^44^ produced on codon-shuffled RTA (CS-RTA)-expressing NIH3T12 cells as previously described^46^ for which JCF and LTK hold a patent [US Patent 11,149,255]. Briefly, CS-RTA4 designed with computer-assisted algorithms eliminating regions of homology to prevent the risk of recombination and WT revertants, was synthesized by OriGene. The ORF50 encoding RTA plasmid, psg5013, had two mutations, a nonsynonymous C to T mutation at nucleotide 242 that was corrected to wild-type and a silent C to T mutation at nucleotide 1225 compared to the reference genome^37^. CS-RTA4 was cloned into the *BgI*II and *Xho*I sites of pMSCV (Murine stem cell virus)-puro (Clontech). MSCV-based retroviral vectors were produced by transfecting BOSC23 cells with either empty pMSCV or pMSCV-RTA constructs using lipofectamine. Two days post-transfection, retroviral supernatants were harvested, filtered, and added directly to NIH 3T12 fibroblasts in culture medium supplemented with 4 μg/ml polybrene. Transduced cells were selected by adding 5 μg/ml puromycin and expanded until puromycin-resistant cells were obtained. ORF50.Stop^44^ MHV68 BACs were transfected into cell lines encoding CS-RTA4 using lipofectamine and PLUS reagent (Invitrogen). Viral supernatants were harvested from transfected cell lysates seven days post-transfection and passaged two additional times on the CS-RTA4 cell line to produce working stocks for experimentation. All viral stocks were harvested by two freeze-thaw cycles followed by centrifugation at 500 g for 10 min at 4 °C to remove cell debris. Viral stocks were concentrated by centrifugation at 35,000 g for 90 min at 4 °C followed by resuspension of virion pellets in the fresh medium of 1/10 original volume. RDV-ORF50.stop recombinant MHV68 produced on CS-RTA4 cell lines was validated by plaque assay on vector control cell lines to demonstrate absence of WT revertants. Heat-inactivated WT MHV68 (WUMS strain) was used as a control intervention group^40^. WT MHV68 was inactivated by heating at 56 °C for 90 min and validated by plaque assay to demonstrate an absence of infectious particles.

### Animal studies

All animal protocols that were performed by Stony Brook University staff were approved by the Institutional Animal Care and Use Committee of Stony Brook University. All animal procedures reported in this study that were performed by NCI-CCR affiliated staff were approved by the NCI Animal Care and Use Committee (ACUC) and in accordance with federal regulatory requirements and standards. All components of the intramural NIH ACU program are accredited by AAALAC International. Mouse experiments were carried out in accordance with guidelines from the National Institute of Health, UAMS Division of Laboratory Animal Medicine (DLAM), and the UAMS Institutional Animal Care and Use Committee (IACUC). The protocols supporting these animal studies were approved by the UAMS IACUC prior to the beginning of the study. Mice were anesthetized prior to inoculation. Mice were assessed twice daily for signs of disease and distress and measured once each day to monitor body weight as an indicator of disease progression. Mice were humanly euthanized at the indicated experimental endpoints or upon displaying signs of distress, characterized by displaying lethargy, dehydration, or a bodyweight reduction of 20% or more from the initial measurement taken on the day of inoculation.

Male and female C57BL/6 mice were purchased from Harlan/Envigo RMS (Indianapolis, IN) Jackson Laboratories (Bar Harbor, ME) or Charles River Laboratories (Wilmington, MA). Male and female *Ifnar1*^*−/−*^ (B6.(Cg)-*Ifnar11tm1.2Ees*/J) mice were ordered from Jackson Laboratories (Bar Harbor, ME). Eight to ten-week-old mice were anesthetized using 1-4% isoflurane in an induction chamber and inoculated with 10^6^ PFU of RDV-50.stop diluted in 200 μL DMEM intraperitoneally and boosted with an equivalent dose on days 25-28 or 39 post-prime. Mock vaccinated and vaccinated C57BL/6 mice were challenged by IN infection with 1 × 10^3^ PFU of WT MHV68 diluted in 20 μl complete DMEM on days 14-17 or 90 post-boost. *Ifnar1*^*−/−*^ mice were challenged with 2x10^6^ PFU WT MHV68, a dose shown previously to be lethal in approximately 50% of cases^63^, on day 16 post-second-boost. At the indicated timepoints, serum was collected by submandibular vein sampling or post-mortem cardiac puncture. Serum, lung, and spleen tissues were harvested following humane euthanasia using isoflurane or CO_2_.

### Pathogenesis assays

For acute titers, mice were euthanized with isoflurane at seven days post-infection, and both lungs were removed and frozen at −80 °C. Lungs were disrupted in 1 ml of 8% cMEM using 1 mm zirconia beads in a bead beater (Biospec, Bartlesville, OK) and plated on NIH 3T12 cells. NIH 3T12 cells were plated at a density of 1.8 x 10^5^ cells/mL one day prior to infection. Serial dilutions of cell homogenate were added to the cell monolayer for 1 hr at 37 °C, with rocking every 15 min, followed by an overlay of 5% methylcellulose (Sigma) in cMEM and incubated at 37 °C. After 7–8 days, cells were fixed with 100% methanol (Sigma) and stained with 0.1% crystal violet (Sigma) in 20% methanol, and plaques were scored.

For limiting dilution analysis, spleens were homogenized in a tenBroek tissue disrupter. Red blood cells were lysed by incubating in 8.3 g/L ammonium chloride for 10 min at room temperature with constant shaking. RBC lysis was neutralized with 25 mL DMEM. Cells were filtered through a 40-micron filter to reduce clumping for further analyses. Limiting-dilution (LD)-PCR analyses to quantify frequencies of latently infected splenocytes were performed as previously described^64^. Briefly, 3-fold serial dilutions of latently infected cells were diluted in a background of uninfected 3T12 fibroblasts. After overnight digestion with proteinase K, cells were subjected to a nested PCR targeting the ORF50 region of the viral genome. Single-copy sensitivity and the absence of false-positive amplicons were confirmed using control standards. Amplicons were visualized for quantitation using ethidium bromide staining in 1.5% agarose gel electrophoresis.

Ex vivo reactivation efficiency was determined as previously described^77^. Briefly, 2-fold serial dilutions of latently infected splenocytes were plated on MEF or BHK21 monolayers for analysis of reactivation from splenocytes from C57BL/6 or *Ifnar1*^*−/−*^, respectively. The presence of preformed infectious virus was detected by plating mechanically disrupted cells on indicator monolayers in parallel. Cytopathic effect was scored 14 and 21 d after plating on MEFs or 6 to 7 d post-infection on BHK21.

### RNA-sequencing

NIH 3T12 fibroblasts were infected with RDV-50.stop or WT MHV68 at MOI 3 for 18 h. Samples were harvested in Trizol, chloroform-extracted, and then RNA was isolated with the Qiagen RNeasy kit including the on-column DNase I treatment. Library preparation and sequencing were conducted at Azenta Life Sciences (South Plainfield, NJ, USA). Extracted RNA samples were quantified using Qubit 2.0 Fluorometer (Life Technologies, Carlsbad, CA, USA) and RNA integrity was checked using Agilent TapeStation 4200 (Agilent Technologies, Palo Alto, CA, USA).

RNA sequencing libraries were prepared using the NEBNext Ultra II RNA Library Prep Kit for Illumina following manufacturer’s instructions (NEB, Ipswich, MA, USA). Briefly, mRNAs were first enriched with Oligo(dT) beads. Enriched mRNAs were fragmented at 94 °C. First-strand and second-strand cDNAs were subsequently synthesized. cDNA fragments were end-repaired and adenylated at 3’ ends, and universal adapters were ligated to cDNA fragments, followed by index addition and library enrichment by limited-cycle PCR. The sequencing libraries were validated on the Agilent TapeStation (Agilent Technologies, Palo Alto, CA, USA), and quantified by using Qubit 2.0 Fluorometer (Invitrogen, Carlsbad, CA) as well as by quantitative PCR (KAPA Biosystems, Wilmington, MA, USA).

RNA-seq data were aligned and counted using the CCR Collaborative Bioinformatics Resource (CCBR) in-house pipeline (https://github.com/CCBR/Pipeliner). Briefly, reads were trimmed of low-quality bases, and adapter sequences were removed using Cutadapt v1.18 (http://gensoft.pasteur.fr/docs/cutadapt/1.18). Mapping of reads to custom reference hybrid genome described below was performed using STAR v2.7.0 f in 2-pass mode^78,79^. Then, RSEM v1.3.0 was used to quantify gene-level expression^80^ with quantile normalization and differential expression of genes analysis performed using limma-voom v3.38.3^81^. The data discussed in this publication have been deposited in NCBI’s Gene Expression Omnibus and are accessible through GEO Series accession GSE227602.

The custom reference genome allowing quantification of both viral and host expression used in this alignment consisted of the mouse reference genome (mm10/Apr. 2019/GRCm38) FASTA with a MHV68 FASTA sequence added as an additional pseudochromosome “GSE227602_mm10_MHV68YFP_Krug.fa.gz” in the GEO accession. This viral genome was prepared from the annotated herpesvirus genome (NCBI reference) with the addition of a CMV-driven Histone H2B-YFP fusion protein locus found in our mutant MHV68 virus strain, utilized to track individually infected cells. The custom gene annotations used for gene expression quantification consisted of a concatenation of the mm10 GENCODE annotation version M21^82^ and annotations of the MHV68 genome. All overlapping regions of the viral ORFs were removed to create a minimal, non-overlapping annotation. The sequencing library used was unstranded, making stranded counts of highly overlapping viral ORFs problematic. This annotation was therefore used to make conservative estimates of the expression of individual viral genes. The custom viral GTF annotation file used for this quantification is “GSE227602_mm10_MHV68YFP_Krug_NoOv.gtf.gz” in the GEO accession.

Data visualizations for RNAseq were created using Prism (GraphPad), and R (https://www.R-project.org/), RStudio (http://www.rstudio.com/), and the pheatmap package (https://CRAN.R-project.org/package=pheatmap)^83^. For both visualizations, counts were normalized first for library size, counts per million mapped reads (CPM), then for composition bias, trimmed-mean of M-values (TMM) before import to RStudio where they were log_2_ transformed prior to heatmap creation using the pheatmap package. For the purposes of visualization, +1 was added to each CPM TMM value prior to log_2_ transformation.

### PCR genotyping

To determine the sensitivity of primers that targeted the FRT locus, we performed a nested PCR reaction on RDV-50.stop BAC DNA using both our designed FRT-targeted primers (dPCR_R1_for GGACCACGCTTTCCAGAGAA, dPCR_R1_rev TCTGGTGGGATGTTGATGGC [805 bp product in RDV-50.stop]; dPCR_R2_for CCATGTGGGTACATCTAGCTTC and dPCR_R2_rev CCAACACATTGCGCCCAAATGTC [308 bp product]), as well as traditional pan-MHV68 primers (KM86 AACTGGAACTCTTCTGTGGC and KM89 GGCCGCAGACATTTAATGAC [586 bp product]; KM87 CCCCAATGGTTCATAAGTGG and KM88 ATCAGCACGCCATCAACATC [382 bp product])^64^. BAC DNA was serially diluted from 10^7^ copies per reaction to 10^−3^ copies per well. Cycling conditions were as previously described^64^. Amplicons were visualized for quantitation. Frequencies of amplification were comparable between FRT-targeted and pan-MHV68 primer pairs.

Nucleic acid was isolated from splenocytes at the conclusion of the study. Total DNA was isolated using a GenCatch blood and tissue genomic mini-prep kit (Epoch Life Science). A nested PCR for the detection of the FRT-scar present in RDV-50.stop MHV68 was performed using 200 ng of genomic DNA with GoTaq polymerase (Promega) using round one primers (dPCR_R1_for and dPCR_R1_rev) and round two primers (dPCR_R2_for and dPCR_R2_rev). Cycling conditions were as previously described^64^. In parallel, a nested PCR was used for the detection of both WT and RDV-50.stop MHV68 was performed using 200 ng of genomic DNA with GoTaq polymerase and pan-MHV68 ORF50 primers as previously described^64^. Amplicons were visualized for quantitation. Frequency of RDV-50.stop MHV68 infection was reported as the percentage of FRT-scar amplicons divided by the total MHV68 ORF50 amplicons.

### Flow cytometry

2 X 10^6^ single cell suspensions prepared from the lung or spleen were resuspended in 200 μl of PBS with 2% fetal bovine serum and blocked with TruStain fcX (BioLegend, San Diego, CA).

T cell subsets were identified with antibodies against CD45 (dilution 1:200; clone 30-F11; BV510; cat# 563891), TCRβ (dilution 1:200; clone H57-597; PerCP-Cy5.5; cat# 109228), CD8 (dilution 1:400; clone 53-6.7; BV785; cat# 100750), CD62L (dilution 1:200; clone MEL-14; FITC; cat# 104406), CD127 (dilution 1:200; clone 678 A7R34; PE-Dazzle594; cat# 135032), and KLRG1 (dilution 1:200; clone 2F1/KLRG1; PE-Cy7; cat# 138416) purchased from BioLegend, San Diego, CA and CD44 (dilution 1:200; clone IM7; APC-eFluor780; cat# 47-0441-82) purchased from eBioscence (Thermo Fisher Scientific). H-2K(b)-p79 or H-2D(b)-p56 MHC-peptide complexes were provided as biotinylated monomers by the NIH tetramer core facility and reconstituted with streptavidin-conjugated APC (used at a dilution of 1:200) or purchased as H-2K(b)-p79 (cat# JD02150) or H-2D(b)-p56 (cat# JA02153) MHC-peptide dextramer complexes conjugated to PE or APC (10 μl of each dextramer added directly to samples) as indicated (Immundex, Fairfax, VA). The data was collected on a CytoFLEX flow cytometer (Beckman Coulter) and analyzed using FlowJoX v10.0.7 (Treestar Inc., Ashland, OR). Cells were first gated as live per exclusion of Alexa Fluor™ 700 NHS Ester uptake and singlet lymphocytes based on forward and side scatter parameters prior to subgating.

### Peptide stimulation

For analysis of T cell effector responses, 1 x 10^6^ splenocytes were plated into each well of a 96-well flat-bottom plate and either left untreated or treated with 1 ug/ml p56 (AGPHNDMEI) or p79 (TSINFVKI) peptides (Genscript, Piscataway, NJ) for 5 h at 37 °C in the presence of Brefeldin A (BD Cytofix/Cytoperm, BD Biosciences). The Fc receptors were blocked prior to surface staining with antibodies against CD45, TCRβ CD8, CD4 (dilution 1:600; clone GK1.5, BV711; cat# 100447; BioLegend), CD4 (dilution 1:200; clone GK1.5; AF488; cat# 100423; BioLegend) and CD44. Upon fixation and permeabilization with the BD Cytofix/Cytoperm kit (BD Biosciences), cells were stained with antibodies to IFNγ (dilution 1:100; clone XMG1.2, PE-Cy7, cat# 505826) and TNFα (dilution 1:100; clone MP6-XT22, BV650, cat# 506333, BioLegend).

### Immunoblot

3T12 fibroblasts were mock-infected or infected with WT MHV68. Lysates were diluted in Laemmli sample buffer and resolved by sodium dodecyl sulfate-polyacrylamide gel electrophoresis (SDS-PAGE). Resolved proteins were transferred to nitrocellulose membranes (Thermo Scientific), and blots were probed with mouse serum harvested from sham and vaccinated animals diluted 1:1000 in 1% milk in TBS with 0.1% Tween-20 (TBS-T). Antibody-bound proteins were recognized using horseradish peroxidase (HRP)-conjugated goat anti-mouse IgG (dilution 1:7500; cat# 115-035-003; Jackson ImmunoResearch). A chemiluminescent signal was detected using a ChemiDoc MP imaging system (Bio-Rad) on blots treated with Clarity ECL reagent (Bio-Rad). All blots were processed in parallel and derive from the same experiments. Unprocessed images are in the Supplementary Information file.

### Enyzme-linked immunosorbent assays (ELISA)

To measure antibody-specificity of serum, high-binding plates (MidSci) were coated with 0.5% paraformaldehyde-fixed viral antigens in carbonate buffer (0.0875 M Na_2_CO_3_, 0.0123 M HCO_3_, pH 9.2) and incubated overnight at 4 °C. Plates were washed in PBS with 0.05% Tween-20 (PBS-T) and blocked with 5% powdered milk-PBS-T for 1 hour at 37 °C. Mouse sera were serially diluted in 1% milk-PBS-T and incubated for 1 hour at 37 °C. IgG was detected by incubation with HRP-conjugated donkey anti-mouse IgG (dilution 1:5000; cat# 715-035-150; Jackson ImmunoResearch). SureBlue substrate (KPL) was added to detect virus-specific antibodies and read at 450 nm on a BioTek Synergy LX plate reader.

### Neutralization assay

Neutralization was tested by means of a plaque reduction neutralization test (PRNT) as described^84^. Briefly, three-fold serum dilutions, starting from an initial concentration of 1:80 in cDMEM were incubated with 150 PFU of MHV68 on ice for one hour. The virus/serum mixture was then added to a sub-confluent BHK21 monolayer (4 x 10^4^ cells/well) plated the previous day in a 24-well plate, in triplicate. As a control, three wells received no-serum added virus. Infected cells were overlaid with methylcellulose and incubated at 37 °C for four days. Methylcellulose media was then aspirated, and cell monolayers were stained with a solution of crystal violet (0.1%) in formalin. Percent neutralization was determined by comparison of the number of plaques in experimental wells compared to no-serum added control wells, and each data point was the average of three wells.

### Statistical analysis

All data was analyzed using GraphPad Prism software (GraphPad Software, http://www.graphpad.com, La Jolla, CA). Frequencies of immune cells analyzed by one-way or two-way ANOVA followed by post-tests depending on parametric distribution. Total numbers of an immune cells subset per animal were log_10_-transformed prior to ANOVA. Titer data were analyzed with unpaired t-test or one-way ANOVA for multiple groups. Based on the Poisson distribution, the frequencies of viral genome–positive cells and reactivation were obtained from the nonlinear regression fit of the data where the regression line intersected 63.2%. Extrapolations were used for samples that did not intersect 63.2%. The log_10_-transformed frequencies of genome-positive cells and reactivation were analyzed by unpaired, two-tailed t-test or one-way ANOVA for multiple groups.

### Reporting summary

Further information on research design is available in the Nature Research Reporting Summary linked to this article.

### Supplementary information

Supplementary Information

Reporting Summary

## Data Availability

The RNAseq datasets generated and/or analyzed during the current study are available in the GEO repository, series accession GSE227602, https://www.ncbi.nlm.nih.gov/geo/query/acc.cgi.
