# Supplementary material for: A replication-deficient gammaherpesvirus vaccine protects mice from lytic disease and reduces latency establishment
Source: NPJ Vaccines. 2024 Jun 24;9:116. doi: 10.1038/s41541-024-00908-x (PMC11196663; doi:10.1038/s41541-024-00908-x)
Supplement: Supplementary file 1 — Supplementary Information [file 41541_2024_908_MOESM1_ESM.pdf]

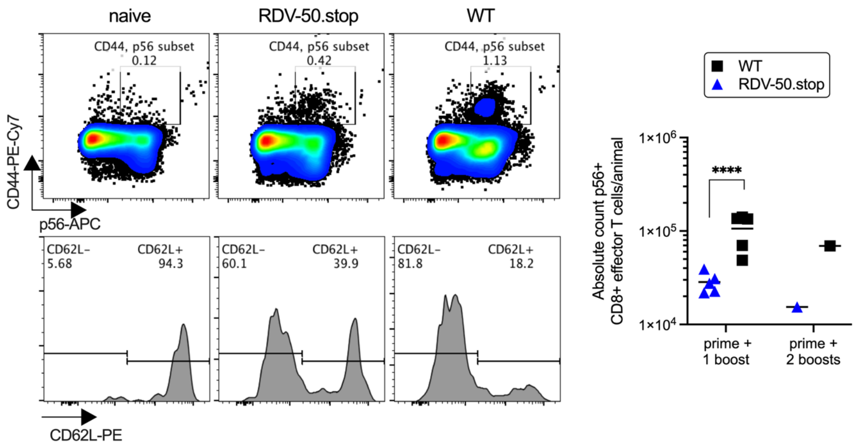

Supplementary Fig 1 Virus-specific CD8 T cell responses upon a prime-boost regimen in C57BL/6 mice. Virus p56-specific effector CD8-T cell response based on p56 tetramer+ of CD44^{hi}CD62L^{-} CD8 T cells at d14 post prime-boost with RDV-50.stop. Naïve mice were age-matched, non-vaccinated controls. (Left) Representative gating strategy from naïve mice or mice infected with RDV-50.stop or WT virus. (Right) Total p56-reactive CD8 T cells per spleen of individual mice after initial prime and sequential boosts. Symbols represent individual mice (N=5) for boost 1 and bars are mean values; ****, p<0.0001 in unpaired t test.

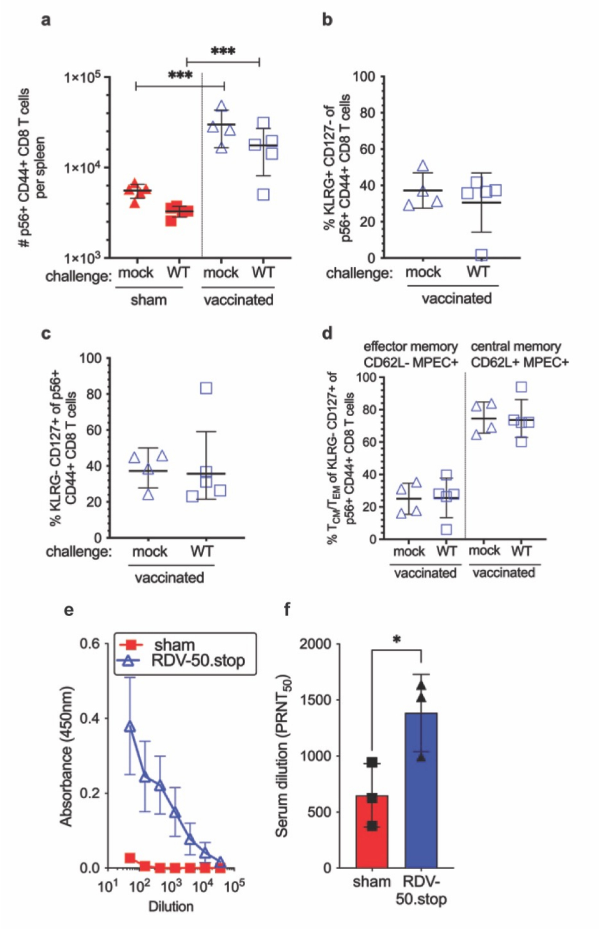

Supplementary Fig 2 Evaluation of immune response to MHV68 at seven days post-challenge with WT virus. C57BL/6 mice were either sham-vaccinated or vaccinated twice (prime+boost) with 1x10^{6} PFU RDV-50.stop followed by mock challenge or challenge with 1x10^{3} PFU WT MHV68 at d15 post-boost and analyzed d7 post-challenge. **a** Total number of CD44^{hi} CD8 T cells in the spleen that were reactive with the viral p56 epitope. **b** p56-tetramer+ CD8 T cells were further analyzed for markers of short-lived effector cells (SLEC, KLRG^{+}CD127^{-}) and **c** memory precursor effector cell subsets (MPEC, KLRG^{-}CD127^{+}). **d** MPECs were further delineated into CD62L^{-} effector and CD62L^{+} central MPECs. For **a-d**, symbols represent individual mice, (N=4-5); bars and whiskers are mean +/- SD. *, p<0.05; ***, p<0.001; ****, p<0.0001 in Sidak's multiple comparisons test of one-way ANOVA between the indicated groups. **e** Virus-specific IgG from sham or RDV-50.stop vaccinated mice at d7 post-challenge measured by ELISA. **f** Virus neutralization in serum as determined by a plaque reduction assay. The PRNT$_{50}$ value is the dilution of serum to reach 50% neutralization of plaques. Symbols represent individual mice (N=3-5); bars are mean +/- SD. *, p<0.05 in unpaired t test.

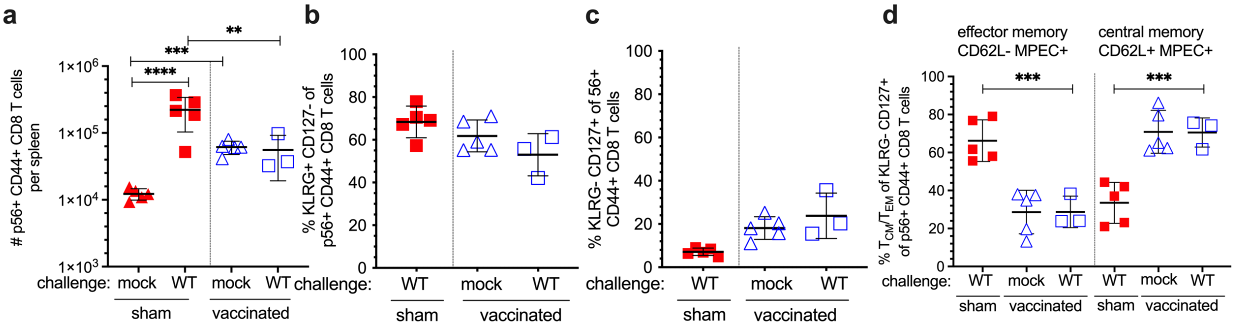

Supplementary Fig 3 Evaluation of the T cell response to MHV68 p56 in vaccinated mice at 16 days post-challenge with WT virus. C57BL/6 mice were either sham-vaccinated or vaccinated twice (prime+boost) with $1x10^6$ PFU RDV-50.stop MHV68 followed by challenge with $1x10^3$ PFU WT MHV68 at d15 post-boost. **a** Total p56-tetramer+ CD8 T cells per spleen of individual mice after initial prime and sequential boosts. Percentage of p56-tetramer+ CD8 T cells with markers of **b** short-lived effector cell (SLEC, $KLRG^+CD127^-$) and **c** memory precursor effector cell subsets (MPEC, $KLRG^-CD127^+$). **d** MPECs were further delineated into $CD62L^-$ effector and $CD62L^+$ central MPECs for p79- and p56-tetramer+ CD8 T cells. For each graph, symbols represent individual mice, (N=3-5); bars and whiskers are mean +/- SD. *p<0.05; ***, p<0.001; ****, p<0.0001 in Sidak's multiple comparisons test of one-way ANOVA between the indicated groups.

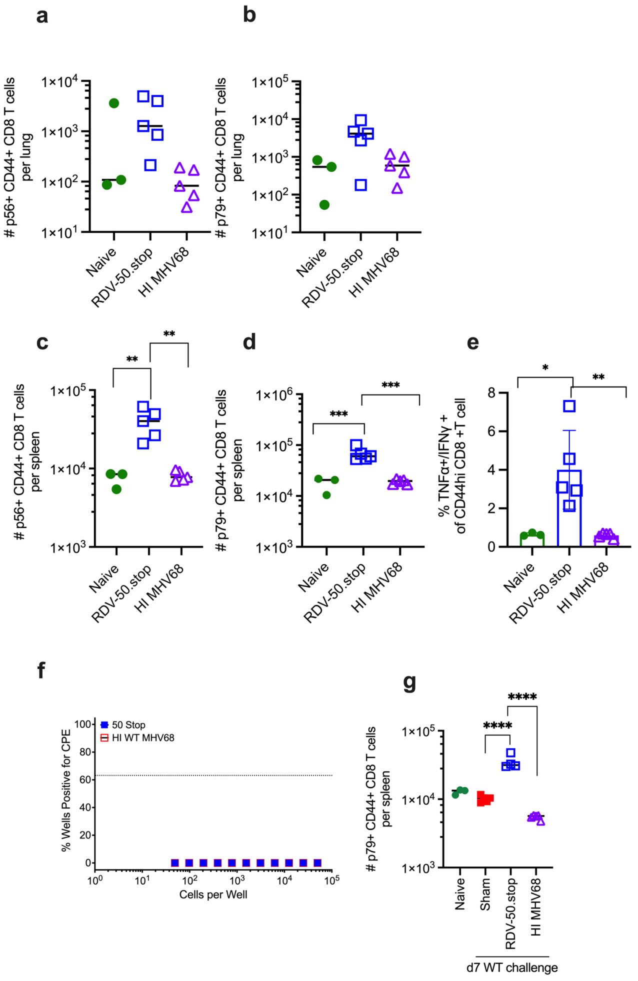

Supplementary Fig 4 RDV-50.stop demonstrates greater efficacy when compared to a heat-inactivated vaccine. C57BL/6 mice were either sham-vaccinated or vaccinated (prime+boost) with 1x106 PFU RDV-50.stop or heat-inactivated (HI) WT MHV68 **a** Total p56-tetramer+ and **b** p79-tetramer+ CD8 T cells per lung of individual mice at d31 post-boost. **c** Total p56-tetramer+ and **d** p79-tetramer+ CD8 T cells per spleen of individual mice at d31 post-boost. **e** Percentage of CD44hi CD8 T cells producing both TNFα and IFNγ in response to viral p56 and p79 peptide dual stimulation at d31 post-boost. For **a-e,** symbols denote individual mice (N=5); bars and whiskers represent mean +/- SD. *, $p<0.05$; **, $p<0.001$; ***, $p<0.0005$; in Sidak's multiple comparisons test of one-way ANOVA between the indicated groups. **f** The frequency of explant reactivation determined by limiting dilution coculture of intact viable splenocytes on a monolayer of primary MEFs d31 post-boost. Disrupted splenocytes plated in parallel did not detect preformed infectious virus in the vaccinated animals. Following prime+boost vaccination, mice were challenged with 1x103 PFU WT MHV68 at d14 post-boost. **g** Total p79-tetramer+ CD8 T cells per spleen of individual mice at d7 post-challenge. Symbols denote individual mice (N=5); bars and whiskers represent mean +/- SD. ****, $p<0.0001$; in Sidak's multiple comparisons test of one-way ANOVA.

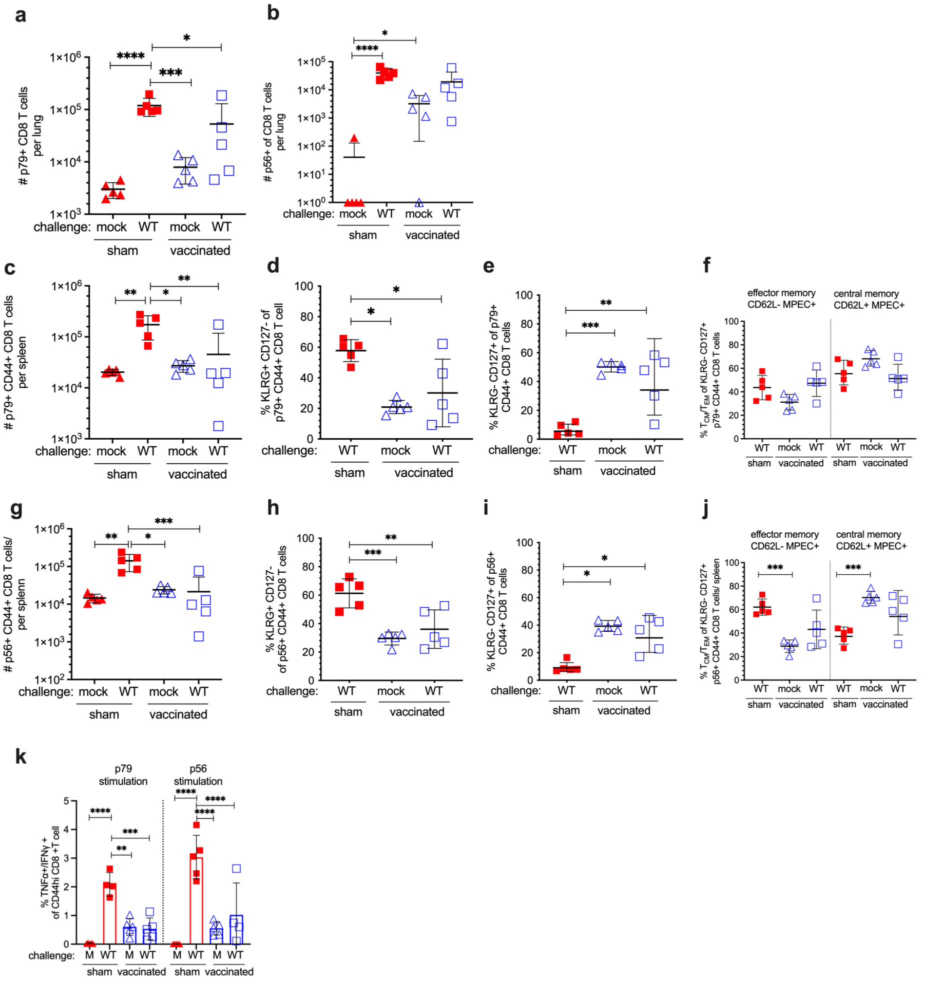

Supplementary Fig 5 Vaccination with RDV-50.stop leads to durable protection against wild-type MHV68 challenge. C57BL/6 mice were either sham-vaccinated or vaccinated twice (prime+boost) with 1x10^6 PFU RDV-50.stop followed by challenge with 1x10^3 PFU WT MHV68 at d90 post-boost, then analyzed d16 post-challenge. **a-b** Total p79- or p56-tetramer+ CD8 T cells per lung. **c,g** Total p79- or p56-tetramer+ CD8 T cells per spleen. Tetramer+ CD8 T cells were further analyzed for markers of **d,h** short-lived effector cell (SLEC, $KLRG^{+}CD127^{-}$) and **e,i** memory precursor effector cell subsets (MPEC, $KLRG^{-}CD127^{+}$). **f,j** MPECs were further delineated into CD62L- effector and CD62L+ central MPECs. **k** Percentage of $CD44^{hi}$ CD8 T cells producing both TNFα and IFNγ after peptide stimulation. For each graph, symbols represent individual mice, (N=4-5); bars and whiskers are mean +/- SD. p<0.05; **, p<0.05; ***, p<0.001; ****, p<0.0001 in Sidak's multiple comparisons test of one-way ANOVA between the indicated groups.

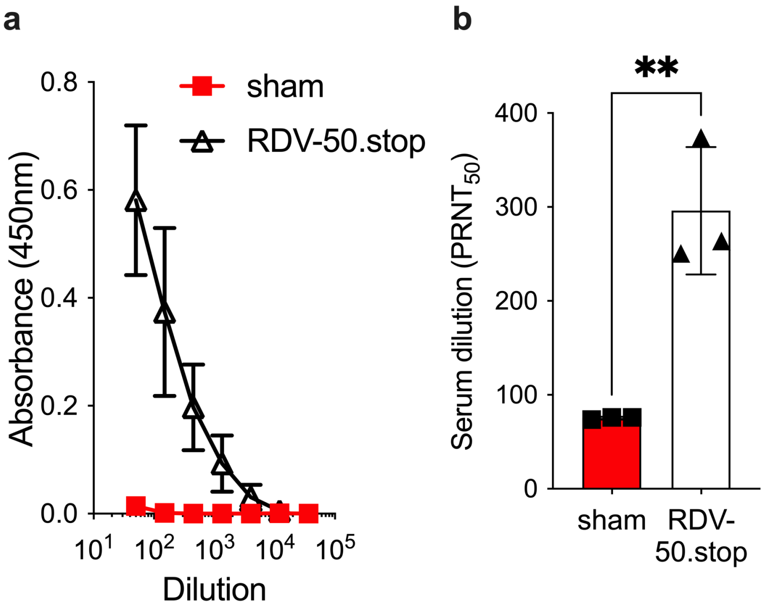

Supplementary Fig 6 *Ifnar1$^{-/-}$* mice generate neutralizing humoral immune responses following RDV-50.stop vaccination. *Ifnar1$^{-/-}$* mice were primed and then boosted twice with $1x10^6$ PFU of RDV-50.stop in the peritoneum, and serum was collected on d52 post-vaccination. **a** Virus-specific IgG from sham or RDV-50.stop vaccinated mice measured by ELISA. **b** Virus neutralization in serum as determined by plaque assay. The $PRNT_{50}$ value is the dilution of serum to reach 50% neutralization of plaques. Symbols represent pooled serum run in technical triplicate. Error bars represent standard error of the means. **, $p<0.005$ as determined by an unpaired, two-tailed student's t test.

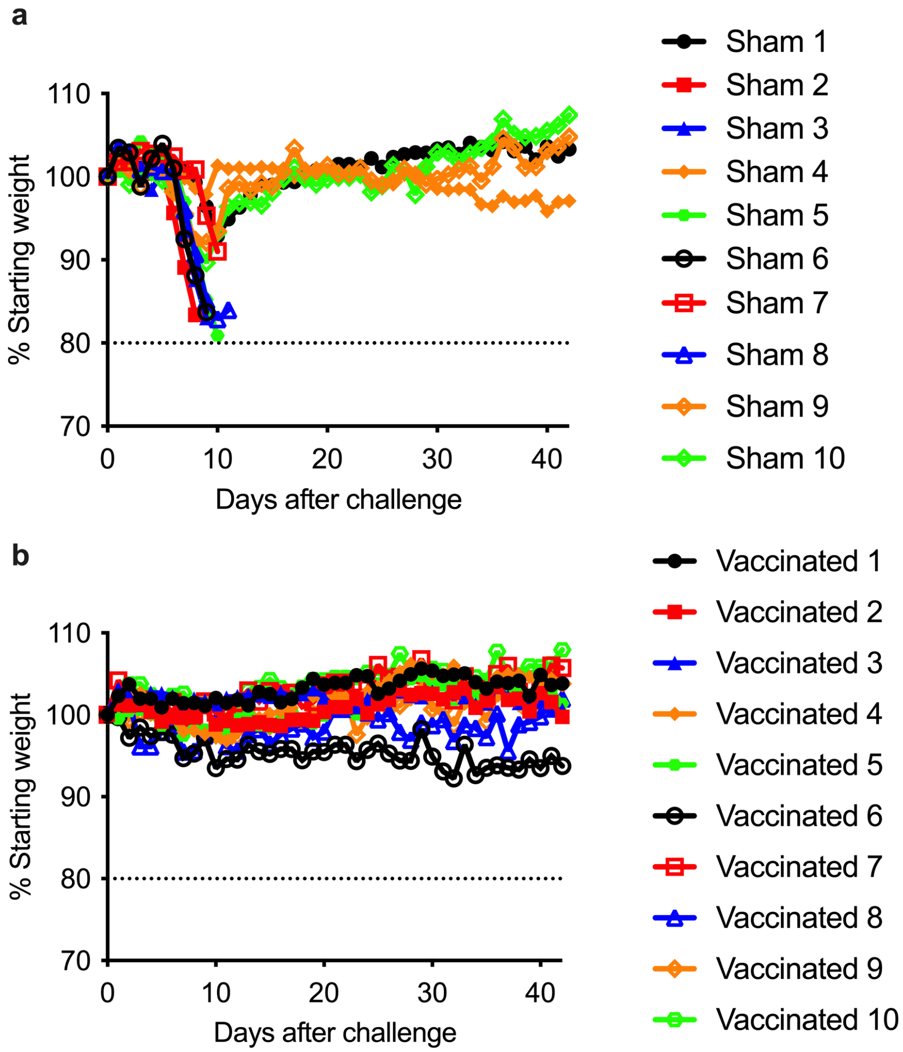

Supplementary Fig 7 Vaccination with RDV-50.stop MHV68 protects mice susceptible to severe disease from weight-loss. *Ifnar1*$^{-/-}$ mice (n=10) were either sham-vaccinated or vaccinated with 3 doses of 1×10^6 PFU RDV-50.stop MHV68 and challenged with a lethal dose of 2×10^6 PFU WT MHV68. **a** Sham-vaccinated and **b** vaccinated mice were weighed daily for 42 days to measure disease progression. Symbols represent individual mice; error bars represent standard error of the means.

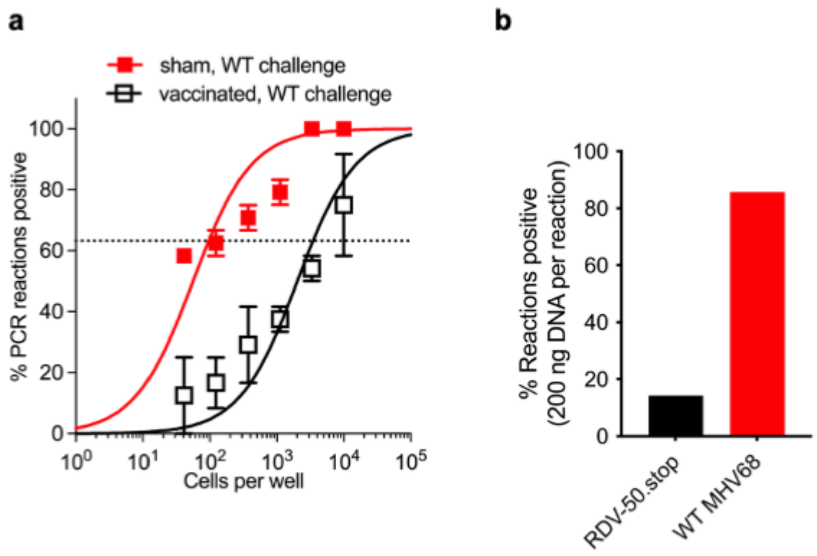

Supplementary Fig 8 RDV-50.stop establishes latency and does not induce sterilizing immunity in *Ifnar1*$^{-/-}$ mice. *Ifnar1*$^{-/-}$ mice were either sham-vaccinated or vaccinated with 3 doses of $1x10^6$ PFU RDV-50.stop and challenged with a lethal dose of $2x10^6$ PFU WT MHV68. **a** The frequency of latency determined by limiting dilution nested PCR of intact splenocytes for the viral genome at d20 post-challenge. **b** PCR genotyping of a pool of splenocytes from RDV-50.stop vaccinated and challenged mice (N=10) at d20 post-challenge. To differentiate the RDV-50.stop vaccine virus from WT challenge virus, nested PCR was performed with primers that target the FRT sequence only present within RDV-50.stop, in parallel with 'pan-MHV68' primers that detect both RDV-50.stop and WT MHV68. For each set, bars indicate the percentage of PCR reactions that produced RDV-50.stop FRT amplimers as a percentage of reactions that produced pan-MHV68 amplimers. The absence of RDV-50.stop FRT amplimers in samples that yielded pan-MHV68 amplimers was considered WT.

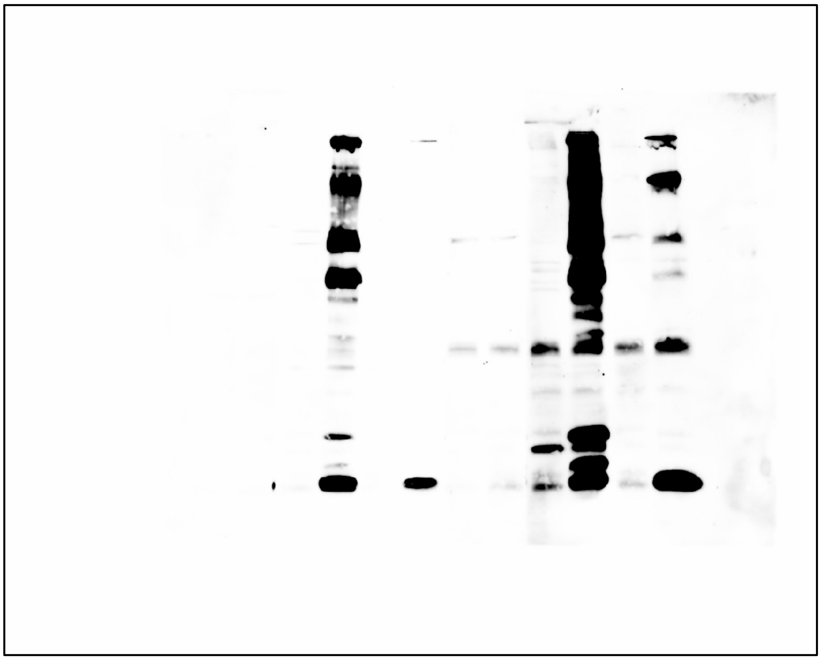

Unprocessed image for Fig 1E to examine serum reactivity.

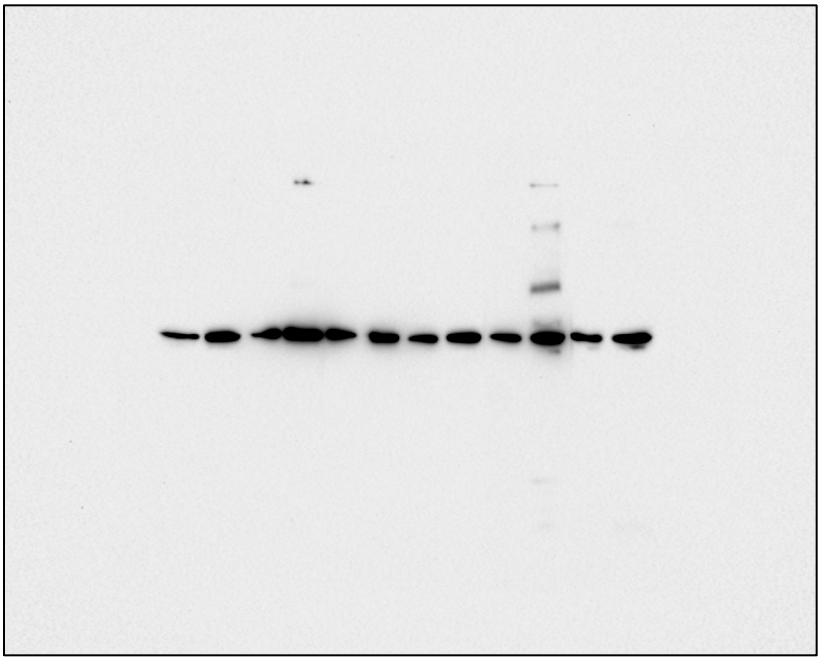

Unprocessed image for Fig 1E to examine actin as loading control.
