# Supplementary material for: A replication-deficient gammaherpesvirus vaccine protects mice from lytic disease and reduces latency establishment
Source: NPJ Vaccines. 2024 Jun 24;9:116. doi: 10.1038/s41541-024-00908-x (PMC11196663; doi:10.1038/s41541-024-00908-x)
Supplement: Supplementary file 2 — Reporting Summary [file 41541_2024_908_MOESM2_ESM.pdf]

nature portfolio

Corresponding author(s): Laurie T. Krug

Last updated by author(s): Apr 17, 2024

Reporting Summary

Nature Portfolio wishes to improve the reproducibility of the work that we publish. This form provides structure for consistency and transparency in reporting. For further information on Nature Portfolio policies, see our Editorial Policies and the Editorial Policy Checklist.

Statistics

For all statistical analyses, confirm that the following items are present in the figure legend, table legend, main text, or Methods section.

n/a	Confirmed	
☐	☒	The exact sample size (*n*) for each experimental group/condition, given as a discrete number and unit of measurement
☐	☒	A statement on whether measurements were taken from distinct samples or whether the same sample was measured repeatedly
☐	☒	The statistical test(s) used AND whether they are one- or two-sided<br>*Only common tests should be described solely by name; describe more complex techniques in the Methods section.*
☐	☒	A description of all covariates tested
☐	☒	A description of any assumptions or corrections, such as tests of normality and adjustment for multiple comparisons
☐	☒	A full description of the statistical parameters including central tendency (e.g. means) or other basic estimates (e.g. regression coefficient) AND variation (e.g. standard deviation) or associated estimates of uncertainty (e.g. confidence intervals)
☒	☐	For null hypothesis testing, the test statistic (e.g. *F*, *t*, *r*) with confidence intervals, effect sizes, degrees of freedom and *P* value noted<br>*Give P values as exact values whenever suitable.*
☒	☐	For Bayesian analysis, information on the choice of priors and Markov chain Monte Carlo settings
☒	☐	For hierarchical and complex designs, identification of the appropriate level for tests and full reporting of outcomes
☒	☐	Estimates of effect sizes (e.g. Cohen's *d*, Pearson's *r*), indicating how they were calculated

Our web collection on statistics for biologists contains articles on many of the points above.

Software and code

Policy information about availability of computer code

Data collection	*Provide a description of all commercial, open source and custom code used to collect the data in this study, specifying the version used OR state that no software was used.*
Data analysis	GraphPad Prism software (GraphPad Software, http://www.graphpad.com, La Jolla, CA) was used for all data analysis.<br>For RNAseq, reads were trimmed of low-quality bases and adapter sequences were removed using Cutadapt v1.18 (http://gensoft.pasteur.fr/docs/cutadapt/1.18). Mapping of reads to custom reference hybrid genome described below was performed using STAR v2.7.0f in 2-pass mode77,78. Then, RSEM v1.3.0 was used to quantify gene-level expression79 with quantile normalization and differential expression of genes analysis performed using limma-voom v3.38.380. The custom reference genome allowing quantification of both viral and host expression used in this alignment consisted of the mouse reference genome (mm10/Apr. 2019/GRCm38) FASTA with a MHV68 FASTA sequence added as an additional pseudochromosome "GSE227602_mm10_MHV68YFP_Krug.fa.gz" in the GEO accession. The data discussed in this publication have been deposited in NCBI's Gene Expression Omnibus and are accessible through GEO Series accession GSE227602. Data visualizations for RNAseq were created using Prism (GraphPad), and R (https://www.R-project.org/)82, RStudio (http://www.rstudio.com/)83, and the pheatmap package (https://CRAN.R-project.org/package=pheatmap)84.

For manuscripts utilizing custom algorithms or software that are central to the research but not yet described in published literature, software must be made available to editors and reviewers. We strongly encourage code deposition in a community repository (e.g. GitHub). See the Nature Portfolio guidelines for submitting code & software for further information.

Data

Policy information about availability of data

All manuscripts must include a data availability statement. This statement should provide the following information, where applicable:

- Accession codes, unique identifiers, or web links for publicly available datasets
- A description of any restrictions on data availability
- For clinical datasets or third party data, please ensure that the statement adheres to our policy

All data supporting the findings of this study are available within the paper and its Supplementary Information. The RNAseq data discussed in this publication have been deposited in NCBI's Gene Expression Omnibus and are accessible through GEO Series accession GSE227602.

Research involving human participants, their data, or biological material

Policy information about studies with human participants or human data. See also policy information about sex, gender (identity/presentation), and sexual orientation and race, ethnicity and racism.

Reporting on sex and gender	*Use the terms sex (biological attribute) and gender (shaped by social and cultural circumstances) carefully in order to avoid confusing both terms. Indicate if findings apply to only one sex or gender; describe whether sex and gender were considered in study design; whether sex and/or gender was determined based on self-reporting or assigned and methods used. Provide in the source data disaggregated sex and gender data, where this information has been collected, and if consent has been obtained for sharing of individual-level data; provide overall numbers in this Reporting Summary. Please state if this information has not been collected. Report sex- and gender-based analyses where performed, justify reasons for lack of sex- and gender-based analysis.*
Reporting on race, ethnicity, or other socially relevant groupings	*Please specify the socially constructed or socially relevant categorization variable(s) used in your manuscript and explain why they were used. Please note that such variables should not be used as proxies for other socially constructed/relevant variables (for example, race or ethnicity should not be used as a proxy for socioeconomic status). Provide clear definitions of the relevant terms used, how they were provided (by the participants/respondents, the researchers, or third parties), and the method(s) used to classify people into the different categories (e.g. self-report, census or administrative data, social media data, etc.) Please provide details about how you controlled for confounding variables in your analyses.*
Population characteristics	*Describe the covariate-relevant population characteristics of the human research participants (e.g. age, genotypic information, past and current diagnosis and treatment categories). If you filled out the behavioural & social sciences study design questions and have nothing to add here, write "See above."*
Recruitment	*Describe how participants were recruited. Outline any potential self-selection bias or other biases that may be present and how these are likely to impact results.*
Ethics oversight	*Identify the organization(s) that approved the study protocol.*

Note that full information on the approval of the study protocol must also be provided in the manuscript.

Field-specific reporting

Please select the one below that is the best fit for your research. If you are not sure, read the appropriate sections before making your selection.

☐ Life sciences ☐ Behavioural & social sciences ☐ Ecological, evolutionary & environmental sciences

For a reference copy of the document with all sections, see nature.com/documents/nr-reporting-summary-flat.pdf

Life sciences study design

All studies must disclose on these points even when the disclosure is negative.

Sample size	Determination of sample sizes were based on previous experience and existing literature with the MHV68 model system
Data exclusions	We did not exclude data in this study.
Replication	For the majority of experiments, we performed two independent experiments and included all the data points without exclusion.
Randomization	Randomization was not applied in this study.
Blinding	*Describe whether the investigators were blinded to group allocation during data collection and/or analysis. If blinding was not possible, describe why OR explain why blinding was not relevant to your study.*

Behavioural & social sciences study design

All studies must disclose on these points even when the disclosure is negative.

Study description	*Briefly describe the study type including whether data are quantitative, qualitative, or mixed-methods (e.g. qualitative cross-sectional, quantitative experimental, mixed-methods case study).*
Research sample	*State the research sample (e.g. Harvard university undergraduates, villagers in rural India) and provide relevant demographic information (e.g. age, sex) and indicate whether the sample is representative. Provide a rationale for the study sample chosen. For studies involving existing datasets, please describe the dataset and source.*
Sampling strategy	*Describe the sampling procedure (e.g. random, snowball, stratified, convenience). Describe the statistical methods that were used to predetermine sample size OR if no sample-size calculation was performed, describe how sample sizes were chosen and provide a rationale for why these sample sizes are sufficient. For qualitative data, please indicate whether data saturation was considered, and what criteria were used to decide that no further sampling was needed.*
Data collection	*Provide details about the data collection procedure, including the instruments or devices used to record the data (e.g. pen and paper, computer, eye tracker, video or audio equipment) whether anyone was present besides the participant(s) and the researcher, and whether the researcher was blind to experimental condition and/or the study hypothesis during data collection.*
Timing	*Indicate the start and stop dates of data collection. If there is a gap between collection periods, state the dates for each sample cohort.*
Data exclusions	*If no data were excluded from the analyses, state so OR if data were excluded, provide the exact number of exclusions and the rationale behind them, indicating whether exclusion criteria were pre-established.*
Non-participation	*State how many participants dropped out/declined participation and the reason(s) given OR provide response rate OR state that no participants dropped out/declined participation.*
Randomization	*If participants were not allocated into experimental groups, state so OR describe how participants were allocated to groups, and if allocation was not random, describe how covariates were controlled.*

Ecological, evolutionary & environmental sciences study design

All studies must disclose on these points even when the disclosure is negative.

Study description	*Briefly describe the study. For quantitative data include treatment factors and interactions, design structure (e.g. factorial, nested, hierarchical), nature and number of experimental units and replicates.*
Research sample	*Describe the research sample (e.g. a group of tagged Passer domesticus, all Stenocereus thurberi within Organ Pipe Cactus National Monument), and provide a rationale for the sample choice. When relevant, describe the organism taxa, source, sex, age range and any manipulations. State what population the sample is meant to represent when applicable. For studies involving existing datasets, describe the data and its source.*
Sampling strategy	*Note the sampling procedure. Describe the statistical methods that were used to predetermine sample size OR if no sample-size calculation was performed, describe how sample sizes were chosen and provide a rationale for why these sample sizes are sufficient.*
Data collection	*Describe the data collection procedure, including who recorded the data and how.*
Timing and spatial scale	*Indicate the start and stop dates of data collection, noting the frequency and periodicity of sampling and providing a rationale for these choices. If there is a gap between collection periods, state the dates for each sample cohort. Specify the spatial scale from which the data are taken*
Data exclusions	*If no data were excluded from the analyses, state so OR if data were excluded, describe the exclusions and the rationale behind them, indicating whether exclusion criteria were pre-established.*
Reproducibility	*Describe the measures taken to verify the reproducibility of experimental findings. For each experiment, note whether any attempts to repeat the experiment failed OR state that all attempts to repeat the experiment were successful.*
Randomization	*Describe how samples/organisms/participants were allocated into groups. If allocation was not random, describe how covariates were controlled. If this is not relevant to your study, explain why.*
Blinding	*Describe the extent of blinding used during data acquisition and analysis. If blinding was not possible, describe why OR explain why blinding was not relevant to your study.*

Did the study involve field work? ☐ Yes ☐ No

Field work, collection and transport

Field conditions	*Describe the study conditions for field work, providing relevant parameters (e.g. temperature, rainfall).*
Location	*State the location of the sampling or experiment, providing relevant parameters (e.g. latitude and longitude, elevation, water depth).*
Access & import/export	*Describe the efforts you have made to access habitats and to collect and import/export your samples in a responsible manner and in compliance with local, national and international laws, noting any permits that were obtained (give the name of the issuing authority, the date of issue, and any identifying information).*
Disturbance	*Describe any disturbance caused by the study and how it was minimized.*

Reporting for specific materials, systems and methods

We require information from authors about some types of materials, experimental systems and methods used in many studies. Here, indicate whether each material, system or method listed is relevant to your study. If you are not sure if a list item applies to your research, read the appropriate section before selecting a response.

Materials & experimental systems

n/a	Involved in the study
☐	☒ Antibodies
☐	☒ Eukaryotic cell lines
☒	☐ Palaeontology and archaeology
☐	☒ Animals and other organisms
☒	☐ Clinical data
☒	☐ Dual use research of concern
☒	☐ Plants

Methods

n/a	Involved in the study
☒	☐ ChIP-seq
☐	☒ Flow cytometry
☒	☐ MRI-based neuroimaging

Antibodies

Antibodies used	Anti-CD45 (clone 30-F11, BV510, cat# 563891, BioLegend); Anti-TCR (clone H57-597, PerCP-Cy5.5, cat# 109228, BioLegend); Anti-CD8 (clone 53-6.7, BV785, cat# 100750, BioLegend); Anti-CD44 (clone IM7, APC-eFluor780, cat# 47-0441-82, eBioscence (Thermo Fisher Scientific); Anti-CD62L (clone MEL-14, FITC, cat# 104406, BioLegend); Anti-CD127 (clone 678 A7R34, PE-Dazzle594, cat# 135032, BioLegend); Anti-KLRG1 (clone 2F1/KLRG1, PE-Cy7, cat# 138416, BioLegend); Anti-CD4 (clone GK1.5, AF488, cat# 100423, BioLegend); Anti-CD4 (clone GK1.5, BV711, cat# 100447, BioLegend); Anti-IFNγ (clone XMG1.2, PE-Cy7, cat# 505826, BioLegend); Anti-TNFα (clone MP6-XT22, BV650, cat# 506333, BioLegend); HRP-conjugated goat anti-mouse IgG (cat# 115-035-003, Jackson ImmunoResearch Laboratories); HRP-conjugated donkey anti-mouse IgG (cat# 715-035-150, Jackson ImmunoResearch Laboratories)
Validation	The target specificities of antibodies purchased from BioLegend, eBioscence (Thermo Fisher Scientific) and Jackson ImmunoResearch Laboratories have been verified by the company.

Eukaryotic cell lines

Policy information about cell lines and Sex and Gender in Research

Cell line source(s)	Primary C57BL/6 murine embryonic fibroblasts (MEFs), NIH 3T12 (ATCC CCL-164), BHK21 (ATCC CCL-10), Vero (ATCC CCL-81), and Vero-Cre cells
Authentication	Cell lines used in this study were not authenticated.
Mycoplasma contamination	Cell lines in this study were tested intermittently for Mycoplasma contamination.
Commonly misidentified lines (See ICLAC register)	No commonly misidentified lines were used in this study.

Animals and other research organisms

Policy information about studies involving animals; ARRIVE guidelines recommended for reporting animal research, and Sex and Gender in Research

Laboratory animals	Male and female C57BL/6 mice were purchased from Harlan/Envigo RMS (Indianapolis, IN) or Jackson Laboratories (Bar Harbor, ME)

Laboratory animals	or Charles River Laboratories (Wilmington, MA). Male and female Ifnar1-/- (B6.(Cg)-Ifnar11tm1.2Ees/J) mice were ordered from Jackson Laboratories (Bar Harbor, ME).
Wild animals	No wild animals were involved in this study
Reporting on sex	Both genders were used for the study of vaccine-mediated protection against lethal challenge in Ifnar1-/- mice.
Field-collected samples	This study did not involve samples collected from the field.
Ethics oversight	All animal protocols that were performed by Stony Brook University staff were approved by the Institutional Animal Care and Use Committee of Stony Brook University. All animal procedures reported in this study that were performed by NCI-CCR affiliated staff were approved by the NCI Animal Care and Use Committee (ACUC) and in accordance with federal regulatory requirements and standards. All components of the intramural NIH ACU program are accredited by AAALAC International. Mouse experiments were carried out in accordance with guidelines from the National Institute of Health, UAMS Division of Laboratory Animal Medicine (DLAM), and the UAMS Institutional Animal Care and Use Committee (IACUC). The protocols supporting these animal studies were approved by the UAMS IACUC prior to the beginning of the study.

Note that full information on the approval of the study protocol must also be provided in the manuscript.

Plants

Seed stocks	*Report on the source of all seed stocks or other plant material used. If applicable, state the seed stock centre and catalogue number. If plant specimens were collected from the field, describe the collection location, date and sampling procedures.*
Novel plant genotypes	*Describe the methods by which all novel plant genotypes were produced. This includes those generated by transgenic approaches, gene editing, chemical/radiation-based mutagenesis and hybridization. For transgenic lines, describe the transformation method, the number of independent lines analyzed and the generation upon which experiments were performed. For gene-edited lines, describe the editor used, the endogenous sequence targeted for editing, the targeting guide RNA sequence (if applicable) and how the editor was applied.*
Authentication	*Describe any authentication procedures for each seed stock used or novel genotype generated. Describe any experiments used to assess the effect of a mutation and, where applicable, how potential secondary effects (e.g. second site T-DNA insertions, mosiacism, off-target gene editing) were examined.*

Flow Cytometry

Plots

Confirm that:

☒ The axis labels state the marker and fluorochrome used (e.g. CD4-FITC).

☒ The axis scales are clearly visible. Include numbers along axes only for bottom left plot of group (a 'group' is an analysis of identical markers).

☒ All plots are contour plots with outliers or pseudocolor plots.

☒ A numerical value for number of cells or percentage (with statistics) is provided.

Methodology

Sample preparation	Single-cell suspensions were obtained from the spleens and lungs were, lysed in ACK (ammoniumchloride-potassium) buffer, and resuspended in PBS with 2% fetal bovine serum, and blocked with TruStain fcX (BioLegend, San Diego, CA). T cell subsets were identified with antibodies against CD45, TCRβ, CD4, CD8, CD44, CD62L, CD127, and KLRG1 along with MHC-peptide dextramer complexes conjugated to PE or APC (Immundex, Fairfax, VA) by incubation at 4 °C for 30 min. For analysis of T cell effector responses, cells were plated into each well of a 96-well flat-bottom plate and either left untreated or treated with 1 ug/ml p56 (AGPHNDMEI) or p79 (TSINFVKI) peptides (Genscript, Piscataway, NJ) for 5 h at 37°C in the presence of Brefeldin A (BD Cytofix/Cytoperm, BD Biosciences). The Fc receptors were blocked prior to surface staining with antibodies against CD45, TCRβ, CD8, CD4, and CD44. Upon fixation and permeabilization with the BD Cytofix/Cytoperm kit (BD Biosciences), cells were stained with antibodies to IFNγ and TNFα.
Instrument	CytoFLEX flow cytometer [CytoFLEX S V4-B2-Y4-R3] (Beckman Coulter)
Software	FlowJoX v10.0.7 (Treestar Inc., Ashland, OR)
Cell population abundance	N/A
Gating strategy	Cells were first gated as live per exclusion of Alexa Fluor™ 700 NHS Ester uptake and singlet lymphocytes based on forward and side scatter parameters prior to subgating. Virus specific T cells were identified by gating on the CD44+CD8+ T cell population and were further characterized by gating on CD127 and KLRG1. KLRG1+CD127- exhibited a short-lived effector cell (SLEC) phenotype and KLRG1-CD127+ exhibited memory precursor effector CD8 T cells (MPEC) phenotype. MPECs were further characterized into CD62L- effector memory and CD62L+ central memory MPECs. Production of antiviral effector cytokines were characterized as TNFα+ IFNγ+ of CD44+CD8+ T cell population.

☐ Tick this box to confirm that a figure exemplifying the gating strategy is provided in the Supplementary Information.
